# Non-Pharmacological Interventions for Managing Apathy in Older Adults with Neurocognitive Disorders: A Systematic Review of Randomized Controlled Trials

**DOI:** 10.3390/brainsci16070687

**Published:** 2026-06-29

**Authors:** Kostas Siarkos, Antonios M. Politis, Anastasios A. Politis, Nikolaos Smyrnis, Charalambos Papageorgiou, Andreas Prentakis, Rossetos Gournellis, Everina Katirtzoglou, Christos Theleritis

**Affiliations:** 1 Second Department of Psychiatry, National and Kapodistrian University of Athens, Attikon Hospital, 12462 Athens, Greece; ksiarkos@med.uoa.gr (K.S.); smyrnis@med.uoa.gr (N.S.); a_prentakis@yahoo.gr (A.P.); rgourn@med.uoa.gr (R.G.); 2 Psychogeriatric Unit, First Department of Psychiatry, National and Kapodistrian University of Athens, Eginition Hospital, 74 Vas. Sofias Ave., 11528 Athens, Greece; apolitis@med.uoa.gr (A.M.P.); evekatir@yahoo.gr (E.K.); 3 Department of Neurosurgery and Neurotraumatology, Athens Medical School, ‘Attikon’ University General Hospital, National and Kapodistrian University, 12462 Athens, Greece; tsspolitis1@gmail.com; 4 Precision Medicine Research Institute “Costas Stefanis”, University Mental Health Neurosciences, 15601 Athens, Greece; chpapag@med.uoa.gr

**Keywords:** apathy, neurocognitive disorders, Alzheimer’s disease, Parkinson’s disease, non-pharmacological interventions, systematic review, randomized controlled trials, exercise therapy, psychosocial intervention, brain stimulation

## Abstract

**Highlights:**

**What are the main findings?**

- Across 62 randomized controlled trials in Alzheimer’s disease and related dementias, Parkinson’s disease, and Huntington’s disease, a range of non-pharmacological interventions—psychosocial, exercise-based, creative, technology-assisted, and brain stimulation—showed potential to reduce apathy, with physical exercise and music-based interventions providing the most consistent support; however, fewer than half of the trials (30/62, 48%) reported a statistically significant between-group benefit, no single class proved superior, and effects were often short-lived.
- Methodological quality was moderate to high, but performance bias was nearly universal owing to the inherent difficulty of blinding non-pharmacological interventions, and marked heterogeneity in apathy assessment, populations, and study designs precluded quantitative synthesis and limited the certainty of the evidence.

**What are the implications of the main findings?**

- A range of non-pharmacological options may help manage apathy in neurocognitive disorders, but their selection should be guided cautiously by disease stage, care setting, and the limited and often short-term evidence available.
- Future trials should treat apathy as a primary outcome using standardized apathy-specific instruments, report effect sizes with precision estimates, and assess the durability of effects to allow quantitative synthesis.

**Abstract:**

Background/Objectives: Apathy is among the most common neuropsychiatric features of late-life neurocognitive disorders and predicts functional decline and greater caregiver burden. As no treatment is formally established, identifying effective interventions is a priority. We systematically reviewed non-pharmacological randomized controlled trials (RCTs) targeting apathy in older adults with neurocognitive disorders. Methods: We searched PubMed/MEDLINE, PsycInfo, the Cochrane Library, and Google Scholar (final search 23 March 2026). Eligible studies were non-pharmacological RCTs reporting an apathy outcome. Evidence levels were graded with OCEBM and quality with PEDro; two reviewers mapped PEDro items onto Cochrane risk-of-bias domains. Reporting followed PRISMA 2020. Results: Sixty-two RCTs were included. Physical exercise and music-based interventions showed the most consistent benefit, whereas technology-based and brain stimulation approaches remained experimental. Only 30 trials (48%) showed a significant between-group effect on apathy—most were null, within-group, or had apathy as a secondary outcome. Marked heterogeneity precluded meta-analysis. Most trials were of moderate to high quality, though near-universal performance bias arose from the inability to blind participants and providers. Conclusions: Managing apathy in these populations remains challenging, and the certainty of the evidence is limited. Purpose-built, apathy-focused trials reporting effect sizes and durability are needed before disease-specific recommendations can be made.

## 1. Introduction

Apathy ranks among the most common and enduring neuropsychiatric features of neurocognitive disorders (NCDs), yet no treatment has been formally established for it. Reported prevalence varies markedly with the underlying condition: roughly 27–72% in Alzheimer’s disease (AD) [1,2,3,4,5], as high as 90% in frontotemporal dementia (FTD), dementia with Lewy bodies (DLB), and progressive supranuclear palsy, about 40% in corticobasal degeneration, and approximately 20% in Parkinson’s disease (PD) [1,6]. The syndrome also emerges after frontal-lobe injury or stroke, where its expression depends substantially on lesion site [6,7]. Apathy can complicate the trajectory and care of dementia and is already detectable at milder stages of cognitive impairment, in both clinic-based [8] and community [5,9] samples, and it has been proposed as one of the earliest behavioral signals of incipient cognitive decline [9]. Its clinical footprint is broad, co-occurring with diminished day-to-day functioning, disability, self-neglect, socially embarrassing conduct, heightened caregiver strain, and a poorer overall prognosis [10,11]. These consequences make the timely recognition and management of apathetic patients a clinical priority.

At the neural level, apathy reflects a breakdown in the prefrontal machinery that normally generates, sustains, and switches goal-directed action [12]. Levy and Dubois [12] attributed this breakdown to three partly dissociable disruptions: an “emotional-affective” component linked to the orbitofrontal cortex and adjacent limbic territory; a “cognitive” component linked to the dorsolateral prefrontal cortex and the caudate nucleus; and an “autoactivation” component linked to the associative and limbic sectors of the globus pallidus. Within dementia specifically, the symptom has been framed as one facet of an executive-dysfunction syndrome arising from injury to frontal–subcortical circuitry [13]. Consistent with this account, apathy severity tracks markers of frontal–subcortical pathology, including neuronal loss, elevated tangle burden, white-matter hyperintensities, and regional hypoperfusion [14,15].

Conceptual definitions have evolved over three decades. Marin [7] originally characterized apathy as a loss of feeling, emotion, interest, concern, or motivation that could not be ascribed to reduced consciousness, cognitive decline, or emotional distress, while Starkstein et al. [15] distilled the construct into three core features—reduced motivation, reduced initiative and interest, and emotional blunting. The consensus criteria operationalized by Robert et al. [16] subsequently reframed apathy as a sustained (≥4 weeks) quantitative decline in goal-directed activity spanning at least two of three dimensions (behavior/cognition, emotion, and social interaction), not better explained by a substance or by environmental change and producing identifiable functional impairment [17]. The most recent diagnostic framework for NCDs [18] preserves this multidimensional structure, requiring persistent or recurrent symptoms over at least four weeks that mark a change from the patient’s usual behavior—encompassing diminished initiative, interest, or emotional expressiveness—that impairs functioning and is not wholly attributable to competing etiologies.

Despite the high prevalence and clinical impact of apathy, no specific treatment has been approved. Nevertheless, a variety of non-pharmacological treatments (NPTs)—spanning psychosocial, exercise-based, technology-assisted, creative, and brain stimulation approaches—have been investigated for the reduction in apathetic symptoms across NCDs, with variable and frequently short-term effects. However, the evidence is characterized by marked heterogeneity in study designs, populations, intervention types, outcome measures, and care settings, which render conventional meta-analytic synthesis problematic. This heterogeneity may increase, in part, by the absence of established diagnostic criteria for apathy and the multidimensional and syndromic nature of apathy itself: clinical apathy implicating motor, cognitive, affective, and behavioral domains suggests that therapeutic benefit may arise from diverse and often complementary treatment classes, and particularly from combinations among them.

In our previous reviews [19,20] we examined the effectiveness of interventions for apathy across treatment modalities; the non-pharmacological interventions, however, were reviewed only in AD populations, while the pharmacological options were addressed separately [21]. The present review differs from this earlier work in three respects: (i) it extends beyond AD to all-cause NCDs (including PD, Huntington’s disease (HD), and mild cognitive impairment (MCI)); (ii) it restricts inclusion to randomized controlled trials (RCTs); and (iii) it incorporates recent trials and newer intervention classes (e.g., immersive virtual reality (VR), social robotics, and non-invasive brain stimulation) that have appeared since our previous syntheses. It is also important to distinguish apathy from confounding states such as depression, fatigue, and cognitive slowing, a distinction that is essential when interpreting studies that relied on broad neuropsychiatric or behavioral scales rather than apathy-specific instruments. Accordingly, the objective of this review is to systematically evaluate the effects of non-pharmacological interventions (the intervention) on apathy severity (the primary outcome) in older adults with NCDs and apathy (the population), compared with usual care, active control, sham, or waitlist conditions, as assessed in RCTs, in order to update the evidence, address methodological issues, inform clinical practice, and guide future research.

## 2. Materials and Methods

### 2.1. Registration and Protocol

The review was not entered in a public registry, and no separate written protocol was produced. Eligibility criteria, search terms, and appraisal instruments were nonetheless specified a priori and applied uniformly, consistent with the methodology of our earlier reviews on related questions [19,20].

### 2.2. Inclusion and Exclusion Criteria

Eligible studies enrolled participants with apathy occurring in the context of a NCD diagnosed by accepted clinical criteria and standardized instruments, delivered a non-pharmacological intervention within a randomized design, and reported at least one apathy-related outcome. Care setting was not a restriction, and neither co-occurring neuropsychiatric symptoms nor concurrent psychoactive medication were treated as an exclusion; systematic reviews were retained for screening purposes. We excluded reports that used a non-randomized or uncontrolled design, supplied no apathy-related outcome, concerned conditions other than an age-related neurodegenerative disorder, and were not published in English. For the narrative synthesis, studies were arranged first by principal NCD (AD and related dementias, PD, HD, MCI) and then, within each, by intervention class (psychosocial and behavioral; music- and art-based; cognitive stimulation; reminiscence- and conversation-based; technology-assisted; physical exercise; multimodal and environmental; and brain stimulation). Throughout, “older adults with neurocognitive disorders” denotes patients diagnosed by recognized criteria (e.g., NINCDS-ADRDA, NIA-AA, DSM, ICD-10, or the Movement Disorder Society criteria for PD). Trials qualified whether apathy was a clinically significant feature at baseline or measured as an outcome; we treat this distinction as a source of heterogeneity and weigh it when interpreting findings. Systematic reviews served only for reference-list checking and contextual discussion and were not themselves synthesized. Randomized crossover trials were eligible, since they incorporate randomization and within-subject control; however, because such designs are vulnerable to carry-over effects—particularly in progressive disorders, where symptoms evolve over time—they were flagged, their results were interpreted as between-condition rather than parallel-group comparisons, and added caution was applied; the two included crossover trials (Kolanowski et al. 2005 [22] and Moyle et al. 2013 [23]) are flagged as such in Table 1. Stable background psychoactive medication was permitted and not treated as a confounder. A study was instead excluded for pharmacological confounding when a pharmacological agent was introduced, withdrawn, or systematically altered as part of the study protocol, or when any change in apathy could not be separated from a concurrent drug effect rather than the non-pharmacological intervention under study. Where a trial did not use a validated apathy-specific instrument, observational or behavioral measures capturing the core apathy construct—diminished goal-directed behavior, lack of initiative, and lack of interest/engagement (e.g., passivity, observed-engagement, or social-withdrawal scales)—were accepted as proxies; reliance on such proxies is treated as a source of measurement heterogeneity and weighed when judging certainty (see Section 4).

### 2.3. Search Strategy and Study Selection

**Figure 1:**
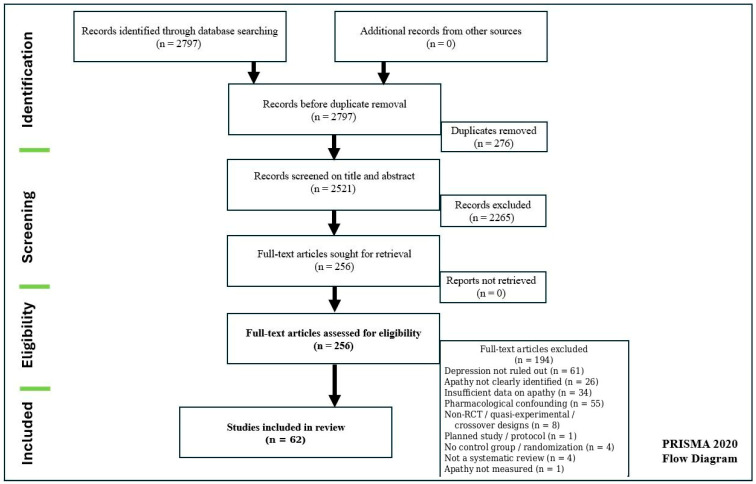
PRISMA flow diagram created using the PRISMA 2020 flow diagram template [85].

The most current search in PubMed/MEDLINE, PsycInfo, the Cochrane Library, and Google Scholar was conducted on 23 March 2026 and the same search strategy was applied in each database. Boolean operators (AND/OR) were used to combine search terms, and an English-language limit was applied; the full search strings for each database are provided as Supplementary Materials, Table S1. Search terms combined diagnostic descriptors with intervention descriptors. Diagnostic terms included apathy, abulia, amotivation, and passivity together with dementia, Alzheimer* disease, FTD, DLB, progressive supranuclear palsy, Parkinson* disease, and Huntington* disease. Intervention and management terms included treatment, management, non-pharmacological, multisensory stimulation (MSS), cognitive stimulation therapy, cognitive rehabilitation, rehabilitation, music therapy, multi-sensory behavior therapy (MSBT)/Snoezelen, physical activity, socialization, reminiscence therapy, art therapy, occupational therapy, tailored activity programs (TAPs), “biography-orientated mobilization” groups, coordinated care intervention, and VR. Titles and abstracts were screened independently by three authors (C.T., K.S., A.M.P.); disagreements were resolved through a consensus discussion involving the senior author. No proprietary extraction template was used; instead, data were entered into a predefined set of fields (participants, diagnosis and diagnostic criteria, intervention, comparator, apathy instrument, direction and statistical significance of the outcome, and quality ratings) and cross-checked between the two reviewers. Every retained article was read in full, with its level of evidence and outcome judged by all authors. Gray and unpublished literature were not sought, and Embase, Scopus, and trial registries (e.g., ClinicalTrials.gov) were not interrogated; we acknowledge this as a limitation. The PRISMA 2020 guidelines [85] were followed for the search strategy, study selection, and reporting (please see flowchart). Because Google Scholar returns large, frequently changing result sets that cannot be reproduced exactly, it was used only as a supplementary source: results were screened in relevance order until successive pages returned no new records. Record counts at each stage—identification, duplicate removal, title/abstract screening, full-text assessment, and exclusions with reasons—are reported in Section 3.1, and the PRISMA 2020 flow diagram (Figure 1) and the full per-database search strings are provided in Supplementary Materials, Table S1.

### 2.4. Data Items

The primary outcome of interest was change in apathy severity, assessed using related tools. Data were sought from all apathy-related measures reported in each study, regardless of whether apathy was designated as a primary or secondary endpoint. Apathy-specific instruments included the Apathy Evaluation Scale in its clinician-administered (AES-C), informant (AES-I), and self-report versions; the Apathy Inventory (AI); the Apathy Scale (Starkstein Apathy Scale, SAS); the Lille Apathy Rating Scale (LARS); the Dementia Apathy Interview and Rating (DAIR); and the Apathy Scale for Institutionalized Patients with Dementia–Nursing Home version (APADEM-NH). Apathy was also captured as a domain-level subscale within broader neuropsychiatric instruments, including the Neuropsychiatric Inventory (NPI) and its variants (NPI-NH, NPI-Q), the Behavioral Pathology in Alzheimer’s Disease scale (BEHAVE-AD), the Behavior Observation Scale for Psychogeriatric Inpatients (BIP), and the Multidimensional Observational Scale for Elderly Subjects (MOSES). Additional behavioral and observational measures from which apathy-relevant data were extracted included the Passivity in Dementia Scale (PDS), the Observed Emotion Rating Scale (OERS), the Philadelphia Geriatric Center Affect Rating Scale (ARS/PARS), the Pearson Environment Apathy Rating (PEAR), and the Social Engagement Scale (SES) (please also see Table 1). Where multiple apathy measures were reported within a single study, all compatible results were extracted. No restriction was placed on the timing of outcome assessment; both post-intervention and follow-up time points were recorded when available.

Beyond apathy outcomes, data were extracted on participant characteristics including sample size, age, sex distribution, neurocognitive diagnosis and diagnostic criteria used (e.g., NINCDS-ADRDA, NIA-AA, ICD-10, DSM), disease severity as indexed by cognitive screening instruments (e.g., MMSE, MoCA) or staging scales (e.g., Global Deterioration Scale) and care setting (community, outpatient, nursing home, assisted living). Intervention characteristics extracted included the type and theoretical basis of the non-pharmacological intervention, format (individual or group), duration, frequency, and comparator condition (e.g., usual care, active control, sham stimulation, waitlist). Study design features were recorded, including randomization method, blinding status, sample size at randomization and follow-up, and dropout rates. Secondary outcomes were recorded when available and included measures of cognition (e.g., MMSE, MoCA, PANDA), depression severity (e.g., GDS, CSDD, Dementia Mood Picture Test), behavioral and psychological symptoms (e.g., NPI total score, CMAI), quality of life (e.g., QOL-AD, QUALID), anxiety (e.g., RAID), functional status (e.g., Barthel Index, IADL), and caregiver-related outcomes. Funding sources were not systematically extracted. Where information on any variable was missing or unclear from the published report, the data field was recorded as not available. No assumptions were made about missing data, and study investigators were not contacted for additional information.

### 2.5. Synthesis Methods

Narrative synthesis was chosen as the primary approach, given the substantial clinical and methodological heterogeneity across populations, intervention types, comparators, apathy instruments, and the primary-versus-secondary status of apathy. That degree of diversity precluded meaningful pooling of effect estimates, and no statistical synthesis was undertaken.

Studies were grouped first by principal NCD and then by intervention class, and for each we described the direction, magnitude (where reported), and statistical significance of the effect on apathy. No single effect metric was imposed; where available, between-group estimates were extracted in their native form (mean differences, standardized mean differences [SMD], and interaction terms from mixed-model or repeated-measures analyses). Where formal effect sizes were unavailable, between-group *p*-values and within-group pre-post comparisons were taken as the available indicators of effect. Because most trials reported only such *p*-values, and in places only the direction of effect, without standardized or unstandardized effect sizes and confidence intervals, a structured tabulation of apathy effect estimates was infeasible for most studies; estimates were reported in the synthesis wherever individual studies supplied them. This itself underscores the need for trials to report effect sizes with precision estimates, as recommended, for example, by the CONSORT statement [86]. In weighing the evidence, we gave greater emphasis to between-group effects from higher-quality, low-risk-of-bias trials (Physiotherapy Evidence Database [PEDro] [87] score ≥ 7), and flagged within-group, borderline, preventive, and short-term findings for cautious interpretation. Given the heterogeneity of designs and outcomes, conclusions about comparative effectiveness and clinical relevance are necessarily provisional. Relevant reporting standards include the CONSORT extension for non-pharmacological treatments [86] and the Cochrane Risk of Bias 2 tool [88], whose items overlap with the PEDro scale we applied (see below). Certainty of evidence was not appraised with GRADE [89]; the rationale and implications are addressed in the Limitations. To make the synthesis more structured, the evidence for each intervention category was appraised on three explicit dimensions: the consistency of the direction of the effect across trials, the methodological quality of the contributing trials (PEDro score and risk of bias), and whether apathy was a designated primary outcome. Categories supported by several low-risk-of-bias trials (PEDro ≥ 7) with concordant between-group benefits were judged to have the strongest evidence, whereas categories resting on single trials, within-group or secondary-outcome findings, or marked heterogeneity were judged weaker; the resulting category-level summary opens the Discussion (Section 4.1).

Data were neither transformed, standardized, nor converted to a common metric, and missing summary statistics were not imputed; for trials with multiple time points, all available assessments were extracted to capture both immediate and longer-term effects.

Individual study findings are tabulated in two summary tables. Table 1 lists, for each included RCT, the reference, NCD examined, sample size, intervention and comparator conditions, apathy outcome measure(s), and the key findings with the direction and statistical significance of the effect.

Table 2 presents the corresponding PEDro quality scorings [87] and Oxford Center for Evidence-Based Medicine (OCEBM) grades of recommendation [90]; the OCEBM levels-of-evidence framework is provided as Supplementary Materials, Table S4. Studies are organized within the tables by primary neurocognitive disorder category.

A PRISMA 2020 flow diagram (Figure 1) displays the study selection process, reporting the number of records identified through database searching, records screened at the title and abstract stage, full-text articles assessed for eligibility, studies excluded with reasons, and the final number of studies included in the narrative synthesis. Patterns of effectiveness were compared qualitatively across studies within and between intervention classes, considering methodological quality as appraised by PEDro scores and OCEBM levels of evidence. No formal methods were used to explore causes of heterogeneity, and no sensitivity analyses were conducted.

### 2.6. Evaluation

#### 2.6.1. Evidence

Two reviewers (C.T. and K.S.) appraised study quality using two established semi-structured instruments. Each included article was assigned a level of evidence and a corresponding grade of recommendation under the OCEBM framework [90] (Table 1). Randomized trials were additionally scored on the PEDro scale [87], whose eleven items capture: specification of eligibility criteria and source; random allocation; concealed allocation; baseline comparability of groups on key outcomes; participant blinding; blinding of the treating therapists; blinding of at least one outcome assessor; adequate retention (attrition bias); intention-to-treat handling; reporting of between-group statistical comparisons; and reporting of point estimates together with measures of variability. Items 2 to 11 were summed to give a total out of 10, and each trial was graded qualitatively as high (a score of ≥7), moderate (5–6), or poor (≤4) quality.

#### 2.6.2. Risk of Bias

To gauge bias in the included RCTs, we used the PEDro items that overlap with the Cochrane Collaboration’s risk-of-bias tool [88]. PEDro items 2–11 were mapped to the following Cochrane domains: selection bias (random allocation, concealed allocation, baseline similarity), performance bias (blinding of subjects and therapists), detection bias (blinding of at least one outcome assessor), attrition bias (less than 15% dropout, intention-to-treat analysis of at least one key outcome), and reporting bias (between-group comparisons, and point estimates with measures of variability, for at least one key outcome). Two raters (C.T. and K.S.) scored each item independently as met or not met; agreement was very high, with disagreement on a single study resolved by consensus. Per-study, per-domain ratings for all 62 included trials appear in Table 2.

We additionally adopted the systematic-review reporting structure of the Center for Reviews and Dissemination, appraising each study’s methodological quality semi-quantitatively across domains that index potential bias. Measures taken to limit bias in the review process itself included a broad, multi-database search with wide-ranging keywords to reduce omission of relevant studies, pre-specified inclusion and exclusion criteria applied without mid-course protocol changes, standardized appraisal of individual study quality and level of evidence, independent dual-rater scoring across all domains, and screening for duplicate publications to limit bias from multiple reporting.

Reporting bias arising from missing results was not assessed statistically, since the narrative approach and the heterogeneity of outcome measures precluded the quantitative pooling on which such tests depend. Unpublished studies were not sought and registries were not searched for completed-but-unreported trials; these limitations are acknowledged and their implications are discussed below.

## 3. Results

### 3.1. Search Results

Searching ‘apathy’ with ‘neurocognitive disorders’ returned 2787 records, and ‘apathy’ with ‘dementia’ returned 2797. Narrower combinations produced: ‘apathy’ AND ‘neurocognitive disorders’ AND ‘treatment’, 1318; ‘apathy’ AND ‘neurocognitive disorders’ AND ‘non-pharmacological treatment’, 69; ‘apathy’ AND ‘dementia’ AND ‘non-pharmacological treatment’, 87; ‘apathy’ AND ‘Alzheimer’s disease’ AND ‘treatment’, 696; ‘apathy’ AND ‘Lewy bodies dementia’ AND ‘treatment’, 76; ‘apathy’ AND ‘Parkinson’s disease’ AND ‘treatment’, 711; and ‘apathy’ AND ‘Huntington’s disease’ AND ‘treatment’, 76. After removing 276 duplicates and screening 2521 titles and abstracts, 256 full texts were assessed for eligibility, of which 194 were excluded; the categorized exclusion reasons appear in Supplementary Materials, Table S2 and in the PRISMA 2020 flow diagram (Figure 1). Sixty-two RCTs were ultimately included, four of them using neurostimulation (transcranial direct current stimulation or repetitive transcranial magnetic stimulation), two in AD and two in PD (see flow diagram and Table 1). Designs that were not strictly randomized and controlled (e.g., quasi-experimental or stepped-wedge) and one study lacking an apathy-specific outcome were excluded from synthesis; randomized crossover trials, being randomized and controlled, were retained and labeled as such, with potential carry-over considered in their interpretation (Section 2.2). Studies reporting associations between apathy and nutritional status or vascular disease were not treated as intervention trials and were excluded. Every included study carried a control or comparison condition alongside the intervention. The relevant studies are described below.

### 3.2. Alzheimer’s Disease and Related Dementias

A sizeable literature has tested non-pharmacological strategies for apathy across the AD spectrum. Fifteen trials evaluated psychosocial and behavioral approaches, including Simulated Presence, emotion-oriented care, kit-based activity, TAPs, individualized functional training, and coordinated care, with widely varying sample sizes; most were set in nursing homes, enrolled moderate-to-severe Alzheimer’s or mixed dementia, assessed apathy as a secondary domain within broader neuropsychiatric measures, and were of high-to-moderate quality. Seven trials examined music therapy, art therapy, museum-based programs, and art-based storytelling, with varied samples and settings, mostly mild-to-moderate AD, and generally moderate-to-high quality. Five trials addressed cognitive stimulation therapy alone or combined with physical activity and socialization; sample sizes varied substantially, apathy was usually secondary (NPI or AES), quality was mixed, and participant and therapist blinding was uniformly absent. Five trials tested reminiscence therapy, therapeutic conversation, and internet-based reminiscence, predominantly in nursing homes among mild-to-moderate dementia, with variable quality and therapist blinding in two of five. Eight trials evaluated technology-based approaches, immersive and group-based VR, social robots, serious exergames, and tablet-based activation, with apathy primary in half, quality ranging from poor to high, and no participant blinding achieved. Five further AD/dementia trials examined physical exercise, progressive muscle relaxation, and antipsychotic review combined with exercise or social interaction, mostly in nursing homes among moderate-to-severe dementia, at generally moderate-to-high quality with assessor blinding in three of five. Ten trials covered MSS environments, biography-oriented mobilization, doll therapy, gesture–verbal treatment, horticultural therapy, aromatherapy, and pet-assisted interventions, predominantly in nursing home patients with moderate-to-severe dementia and of mixed quality, with apathy primary in fewer than half and no participant blinding in any. A detailed account follows.

Weighed by quality, the strongest dementia evidence comes from trials scoring high on PEDro (≥7), several of which reported significant between-group reductions in apathy (e.g., music-based and structured psychosocial programs). A comparable number, however, were null or non-significant between groups, and several apparently positive results rested on within-group pre-post change or on apathy assessed only as a secondary domain within broader neuropsychiatric scales, which limits their interpretive weight. Where follow-up was reported, gains frequently faded, with apathy drifting back toward baseline after the intervention ended. These findings are therefore best read as promising but of limited certainty rather than as definitive evidence of efficacy.

Camberg et al. [91] tested Simulated Presence (personalized audiotapes) in 54 nursing home residents with AD and related dementias; it is mentioned for context but, by design, was not counted for as an RCT in this synthesis. Baker et al. [24] compared MSS with one-to-one activity in 33 patients and reported a significant interaction effect on attentiveness to the environment. By contrast, Cott et al. [25] found that a walking/talking program produced no significant group differences in communication, ambulation, or function, even after adjusting for individual variation. Schrijnemaekers et al. [26] randomized 151 residents with cognitive impairment and behavioral problems to emotion-oriented care or control and observed no significant or clinically relevant effect on the apathetic and nonsocial subscales of the GIP. In a later MSS trial, Baker et al. [27] reported that both MSS and control-activity participants engaged more with others and were less bored or inactive after sessions, with severely impaired MSS-group patients showing significantly less apathy on the BRS apathy subscale.

Activity-based and occupational approaches have likewise been studied widely. Politis et al. [28] pitted a kit-based activity program against one-to-one sessions with an activity therapist in 36 patients; apathy eased substantially under both conditions, implying that regular personal contact may itself carry therapeutic value. Chapman et al. [29] reported a Group × Time interaction favoring a cognitive-communication program plus donepezil over donepezil alone on apathy in 54 patients. Lai et al. [30] found no significant between-group difference over time in a three-arm reminiscence RCT, although psychosocial well-being improved within the intervention group (*p* = 0.014). Finnema et al. [32] compared integrated emotion-oriented care with usual care in 146 residents and 99 nursing assistants and reported no significant difference in BIP apathy scores. Kolanowski et al. [22] tested activities derived from the Need-driven Dementia-compromised Behavior model in 30 patients and found significantly less passivity under interest-matched than skill-matched or baseline conditions. A quasi-experimental study by van Weert et al. [92] reported reduced apathetic behavior on the BIP with Snoezelen care; being non-randomized, it is cited for context but was not synthesized. Holmes et al. [33] reported that, relative to silence and pre-recorded music, live interactive music elicited markedly greater engagement in 32 patients. Staal et al. [34] contrasted MSBT with structured activity in 24 participants, concluding that MSBT may relieve apathy beyond what standard care achieves alone. Tadaka and Kanagawa [35] randomized 24 patients and found significant improvement in withdrawal in the reminiscence arm. Gitlin et al. [36] randomized 60 patients to a TAP or waitlist, with caregivers reporting greater activity engagement in the treatment arm; a later outpatient version of the TAP [79] in 54 participants showed a significant within-group reduction in apathy (*p* = 0.02) and in caregiver burden.

Music- and art-based therapies have produced encouraging signals. Raglio et al. [37] found music therapy significantly improved NPI apathy versus control in 59 patients with dementia, and a subsequent cycle-based RCT in 60 patients [42] again showed significant NPI apathy improvement in the music arm. Tang et al. [62] examined group music in 77 nursing home residents, with a significant decrease in AES apathy in the intervention group (z = 4.516, *p* < 0.01) but not the control group (z = −1.810, *p* > 0.05). Ferrero-Arias et al. [43] randomized 146 patients to music-and-art therapy with psychomotor activity versus free activities and found a significant difference on the DAIR scale, especially in patients with moderate apathy. Hattori et al. [44] compared art therapy with calculation training in 39 patients and reported significant improvement on the Apathy Scale in the art arm. Schall et al. [63] tested the ARTEMIS museum-based intervention in 44 dementia patients and their care partners and found significantly lower NPI apathy subscale scores after intervention (t = 2.52; *p* < 0.05). The CrEAS-AC art-based storytelling program [69] was evaluated in 78 patient–caregiver pairs, with significantly lower apathy at 12 and 24 weeks versus social-contact controls (*p* < 0.001).

Cognitive and combined stimulation has been examined in several trials. Niu et al. [41] showed that 10 weeks of cognitive stimulation therapy significantly lowered apathy versus control in 32 AD patients. Maci et al. [46] reported significant AES apathy improvement after a three-month program of cognitive stimulation, physical activity, and socialization in 14 patients. In the multicenter ETNA3 trial, Amieva et al. [50] compared cognitive training, reminiscence therapy, individualized cognitive rehabilitation, and usual care in 653 AD outpatients, with only individualized rehabilitation yielding clinically significant results. A further powered RCT [77] of multi-sensory stimulation in 80 elderly AD patients found significantly lower AES scores after 12 weeks versus routine care (*p* < 0.05).

Reminiscence and conversation-based approaches have been tried in several settings. Tappen and Williams [38] found therapeutic conversation effective for apathy in nursing home residents with AD. Hsieh et al. [39] showed that reminiscence group therapy improved apathy on the AES-Clinician. Inel Manav and Simsek [64] tested internet-based reminiscence in 32 nursing home residents with mild dementia and found ARS total mean scores significantly higher in the intervention group at post-test (*p* < 0.01), with significant within-group improvement (*p* < 0.01) and no change in controls (*p* > 0.05).

Individualized and tailored care has shown variable success. Lam et al. [40] found NPI apathy significantly improved one month after individualized functional training, though gains rebounded by four months. Kolanowski et al. [45] demonstrated, in an RCT, that activities matched to functional level and personality style improved passive behavior on the Passivity in Dementia Scale. A stepped-wedge study by Leontjevas et al. [93] reported reduced apathy (10-item AES) under a depression-management program; given its pseudo-randomized design, it is cited for context but not synthesized. Among technology-based work, Manera et al. [53] found that participants preferred virtual reality over paper-based conditions, with apathetic participants showing a particularly strong VR preference. Valenti Soler et al. [49] reported improved NPI apathy among advanced-dementia patients exposed to social robots. Moyle et al. [23] found, in a pilot cross-over RCT, that a companion robot did not improve AES apathy in 18 patients. Robert et al. [66] evaluated a serious exergame in 91 patients with neurocognitive disorders across 16 centers: apathy (assessed with NPI and AI) fell in the intervention group during training (W12) and remained stable afterward (W24), while a mixed analysis showed a significant increase in controls. Pereira et al. [94] studied immersive VR in a small randomized crossover trial of 20 participants, reporting fewer facial expressions of apathy during immersive sessions (*p* = 0.034); given its crossover design and outcome it is cited for context but not retained among the parallel-group RCTs. A systematic review and meta-analysis of ten VR studies [95] further suggests technology may reduce apathy, though small samples limit confidence. A quasi-experimental group-based VR study [96] reported that apathy diminished significantly across sessions (z = −5.275, *p* < 0.001). The PflegeTab tablet-based intervention [78] in 162 residents showed no significant group difference in apathy reduction (*p* = 0.91) versus conventional activities, although it lowered psychotropic medication use.

Physical exercise and somatic interventions have been examined across care settings. Telenius et al. [47] conducted a single-blind RCT in 163 nursing home residents with dementia and found that intensive strengthening and balance exercise lowered apathy versus control (*p* = 0.048). Ikemata and Momose [52] randomized 44 dementia patients to progressive muscle relaxation or usual activity and found significantly lower NPI-NH apathy together with improved interest, volition, and social relationships in the intervention group. Rajkumar et al. [54] showed, in a cluster RCT, that antipsychotic review combined with social interaction or exercise significantly reduced NPI-NH apathy in nursing home residents. A study by Sampaio et al. [97], linking six months of physical exercise to preserved behavioral stability and lower apathy scores, is cited for context; although outcomes were collected by blinded assessors, the homes and proxy-reporters could not be blinded to allocation and the design was not randomized, so it was not included as an RCT.

Multimodal, environmental, and animal-assisted approaches have produced mixed findings. Sanchez et al. [55] observed a positive effect of an MSS environment on neuropsychiatric symptoms and dementia severity versus an activity group in a pilot RCT. Treusch et al. [48] found, in a 10-month RCT of 117 patients, that the rise in AES apathy was significantly attenuated under biography-oriented mobilization versus controls. Friedmann et al. [60] compared pet-assisted living with reminiscing over 12 weeks in cognitively impaired assisted-living residents and found opposite slopes of change on the seven-item AES, slightly improved for pet-assisted living and slightly worse for reminiscing, though neither change was significant (PAL: *p* = 0.576; reminiscing: *p* = 0.237). Balzotti et al. [61] compared gesture–verbal treatment (GVT) and doll therapy (DT) in 30 dementia patients, with GVT significantly improving NPI apathy versus both controls and DT. Santagata [80] conducted a doll therapy RCT in 52 residents and reported significant global improvement in BPSD, including apathy and delirium incidence. Di Domenico et al. [51] showed, in a case–control study, that a brief emotional-shaping intervention raised immediate motivation in 26 AD patients. Yang et al. [67] examined horticultural therapy in 32 dementia patients, with AES-I apathy at post-intervention (median = 47.5, IQR = 13.0) significantly lower than control (median = 55.5, IQR = 10.0; *p* = 0.007) and baseline (median = 56.5, IQR = 13.0; *p* = 0.032). Li et al. [70] ran a home-based aromatherapy trial in 80 dyads, where lavender oil improved disinhibition and irritability but did not significantly reduce apathy (*p* = 0.310). A pilot RCT of thematic Beach Room sensory stimulation [83] for 49 residents found the standard Grand Cafe control more effective at reducing BPSD than the enriched environment (*p* = 0.91). A Taiwanese cluster RCT [71] of video-based modalities across 16 centers found no significant reduction in apathy severity (*p* = 0.638).

#### Brain Stimulation in Alzheimer’s Disease

Two trials examined non-invasive brain stimulation for apathy in AD. Both were sham-controlled, double-blind, and conducted in outpatients with moderate AD, with apathy being a primary outcome assessed by the AES-C, and both were rated as high-quality, blinding of subjects, therapists, and assessors being feasible through sham protocols. Suemoto et al. [82] randomized 40 moderate-AD patients to active or sham transcranial direct current stimulation (tDCS) over the left dorsolateral prefrontal cortex in a phase II trial; the intervention was well tolerated but produced no significant apathy effect over the two-week period (*p* = 0.552). By contrast, Padala et al. [68] conducted a randomized, double-blind, sham-controlled pilot of rTMS in 20 AD patients with apathy and found significantly greater AES-C improvement with rTMS than sham (−10.1 [−15.9 to −4.3]; t(16) = −3.62; *p* = 0.002) at four weeks.

### 3.3. Parkinson’s Disease

In Parkinson’s disease, the evidence is dominated by exercise- and dance-based interventions. Several trials reported significant improvements in apathy, but many of these were within-group effects observed in a single arm rather than between-group differences, and sample sizes were generally small. The non-invasive brain stimulation trials, although also small, provided the methodologically strongest signals because their sham-controlled designs allowed partial blinding. As in dementia, durability beyond the intervention period was rarely demonstrated, and the overall certainty of the Parkinson’s disease evidence remains limited.

Twelve trials examined apathy interventions in Parkinson’s disease, spanning Nordic walking, dance (ballroom, folk, tango), group and individual exercise, cognitive behavioral therapy, mindfulness, aerobic cycling, and rTMS, at mixed quality and with participant blinding only in the two stimulation trials. Cugusi et al. [56] randomized 20 PD patients to Nordic walking or control for 12 weeks and found a significant between-group difference on the short Starkstein Apathy Scale (*p* < 0.0005): apathy fell in the walking group (22.8 → 16.5) while controls worsened slightly (22.6 → 23.6). Hashimoto et al. [57] assigned 46 mild-to-moderate PD patients to dance, PD exercise, or no intervention and found a significant Group × Time interaction on the Apathy Scale (F(2,42) = 8.0, *p* < 0.05), the dance group improving most (mean AS 14.7 → 10.2) while controls held steady. King et al. [58] randomized 58 PD patients to home, individual, or group exercise; only individual physical therapy significantly improved LARS apathy (*p* < 0.05). Berardelli et al. [59] assigned 20 PD patients with psychiatric comorbidity to CBT or psychoeducation, with AES apathy improving significantly only under CBT. Solla et al. [75] showed, in a pilot RCT, that 12 weeks of Sardinian folk dance prevented the significant worsening seen in controls (*p* = 0.018). Rios Romenets et al. [73] compared Argentine tango with conventional exercise in 33 PD patients and found no significant apathy difference (*p* = 0.904). Sajatovic et al. [74] tested individual and group exercise in 30 PD patients with comorbid depression over 24 weeks; depression improved but apathy did not (*p* = 0.87). Buchwitz et al. [72] evaluated IPSUM mindfulness training in 30 PD participants, where the training group held apathy stable while waitlist controls significantly worsened (*p* = 0.01). Sacheli et al. [81] studied high-intensity aerobic cycling in 35 PD patients; three months increased caudate dopamine release but did not significantly improve apathy versus stretching. Vitale et al. [84] investigated Biodanza SRT in 28 PD participants: motor and cognitive measures improved, but the Time × Group interaction for apathy was non-significant (AES F(1,26) = 3.371; *p* = 0.078). Post hoc tests indicated that controls worsened and scored higher than the intervention group at follow-up (corrected-*p* = 0.025) while the Biodanza group remained stable; given the non-significant interaction, this preventive pattern should be read cautiously rather than as definitive evidence of a treatment effect on apathy. A realist review of nine studies [98] concluded that exercise and mindfulness target apathy in PD through goal-directed behavior change and social engagement, provided cognitive impairment is minimal.

#### Brain Stimulation in Parkinson’s Disease

Oguro et al. [99] gave real versus placebo rTMS over the supplementary motor area to 15 PD patients in a randomized crossover protocol, reporting apathy improvement with real rTMS; given its small crossover design, it is cited for context but not retained among the parallel-group RCTs. Wei et al. [76] applied high-frequency rTMS to 50 PD patients in a placebo-controlled RCT and found a significant reduction in Starkstein Apathy Scale scores versus sham (*p* < 0.0001).

### 3.4. Huntington’s Disease

A single study, by Trinkler et al. [65], examined contemporary dance practiced two hours weekly over five months on motor function, neuropsychiatric variables, cognition, and brain volume in 19 patients with mild-to-moderate HD (TFC range 7–13; UHDRS motor score range 3–58). LARS apathy scores did not differ between patients and controls.

### 3.5. Comprehensive Reviews

A systematic review and meta-analysis [100] of nine RCTs (356 participants) concluded that physical exercise significantly improves apathy in older adults (SMD = −0.32), with low heterogeneity (I^2^ = 14.7%) and, as graded by those authors, high-level evidence; they highlight exercise as a low-risk, high-benefit adjunct to medication that optimizes the dopaminergic basis of motivational decision-making. A meta-analysis of ten virtual reality (VR) studies [95] likewise suggested that VR may reduce apathy alongside aggression and depression in dementia, though the base was limited by the predominance of quasi-experimental designs (only one RCT), small samples, and varied methods. A review of nine PD studies [98] concluded that exercise and mindfulness target apathy through goal-directed behavior change and social engagement, provided cognitive impairment is minimal.

## 4. Discussion

### 4.1. Principal Findings

Interpreting these findings by strength of evidence, the intervention classes with the most consistent support from higher-quality randomized trials are physical exercise and structured music-based interventions, followed by selected psychosocial and multisensory approaches. Technology-based interventions (immersive VR, social robotics, serious exergames) and non-invasive brain stimulation remain promising but should currently be regarded as experimental, given the small samples, heterogeneous protocols, and limited replication. Importantly, several trials that reported initial benefit also showed a rebound effect, with apathy returning toward baseline once the intervention was withdrawn; the absence of durable effects is a recurring limitation across intervention classes. Any clinical “toolkit” of non-pharmacological options must therefore be framed with explicit attention to these short-lived effects and to the need for maintenance strategies.

Translating Table 1 codings into aggregate terms: of the 62 included trials, 30 (48%) reported a statistically significant positive between-group effect on apathy, 21 (34%) of these with apathy as the designated primary outcome and nine (15%) as a secondary or exploratory one; 22 (35%) reported null between-group findings; seven (11%) showed only a within-group pre-post change without a corresponding significant between-group difference; two (3%) used crossover/within-subject designs reported as between-condition comparisons; and one (2%) reported a worsening of apathy. Apathy was a primary outcome in 39 trials (63%) and secondary in 23 (37%). Critically, only two of the 62 trials (3%) assessed apathy beyond the immediate post-intervention endpoint, so the durability of any benefit is essentially uncharacterized across the field. Together, these figures qualify any global claim of efficacy: the headline of “many positive trials” conceals a literature in which roughly half are null or rely on within-group inference, secondary-outcome status is common, and post-intervention follow-up is the exception rather than the rule.

This review set out to synthesize non-pharmacological treatments for apathy across aging neurodegenerative disorders, combined approaches included. Although the magnitude of any genuine effect cannot be quantified here and the designs carry inherent constraints, the aggregated level and quality of the evidence indicate that apathy may improve in these patients under a broad range of interventions—MSS, kit-based activity, cognitive-communication programs, treatment derived from the Need-driven Dementia-compromised Behavior (NDB) model, live interactive music, art therapy, TAPs, individualized occupational therapy, cognitive stimulation therapy, “biography-orientated mobilization” groups, coordinated care interventions, physical exercise, VR, and individualized cognitive rehabilitation, among others. Subsequent evidence has expanded this repertoire, with newer studies demonstrating potential benefits for art-based storytelling programs [69], serious exergames [66], horticultural therapy [67], doll therapy [80], immersive VR (in a small crossover trial) [94], group-based VR [96] and multi-sensory stimulation [77]. A systematic review and meta-analysis [100] synthesizing nine RCTs involving 356 participants reported that physical exercise significantly improves apathy in older adults (SMD = −0.32), positioning exercise as a well-supported adjunctive intervention. Furthermore, group cognitive therapy [50] and rTMS show promising results [68,99] with a newer placebo-controlled trial [76] confirming that high-frequency rTMS significantly reduces apathy in PD (*p* < 0.0001). In contrast, several new trials yielded null findings for apathy at the group level, including a home-based aromatherapy program [70] (*p* = 0.310) for apathy, video-based training modalities [71] (*p* = 0.638), a tablet-based intervention [78] (*p* = 0.91) and a thematic beach-room sensory environment [83].

In PD, mindfulness training [72] held apathy stable while controls worsened (*p* = 0.01), and Sardinian folk dance [75] prevented the worsening seen in controls (*p* = 0.018). However, Argentine tango [73] (*p* = 0.904) and combined exercise and self-management formats [74] (*p* = 0.87) failed to change apathy scores. High-intensity aerobic cycling [81] raised caudate dopamine release but did not translate into apathy improvement, and Biodanza SRT [84] showed a trend toward worsening in controls without a significant between-group difference. For MCI, a cluster RCT of an integrated social-art intervention [101] showed short-term cognitive improvement at 14 weeks that was not sustained at the 24-week follow-up, suggesting that age-related health issues and waning engagement limit long-term efficacy.

More broadly, no eligible RCT reported an apathy-specific outcome in MCI; because apathy may mark progression from MCI to dementia [102], this absence is an important evidence gap and a priority for future research.

Interpreting these findings is further complicated by the overlap between apathy and depression. The two frequently co-occur in NCDs, yet apathy is defined by a primary loss of motivation, initiative, and emotional engagement in the absence of the dysphoria, hopelessness, and negative self-referential cognitions that characterize depression. This distinction matters here because several included trials measured apathy only as a subdomain of broader neuropsychiatric instruments (e.g., the NPI) or alongside depression and engagement scales whose items partially capture amotivation; an apparent effect on apathy may therefore partly reflect change in depressive symptoms, and vice versa. The heterogeneous and frequently non-apathy-specific assessment used across studies thus limits the comparability of outcomes and lowers the certainty with which any intervention can be said to act specifically on apathy. Future trials should pair validated apathy-specific instruments with concurrent depression measures so that the two constructs can be disentangled.

### 4.2. Strengths and Weaknesses

#### 4.2.1. Appraisal of Methodological Quality of the Review

Although a number of trials reported at least a partial benefit of various interventions on apathetic symptoms, many did not designate apathy as their main target, and several relied on measurement tools that were either unvalidated or ill-suited to the construct. Our deliberately pragmatic, real-world framing, while intended to maximize inclusiveness, itself compounds the heterogeneity of the assembled evidence. The studies could only be brought together in a narrative synthesis because they were too dissimilar for pooling. Reported evidence was appraised with two published semi-quantitative instruments. Several limitations apply. First, any conclusion is contingent on the quality of the primary studies, and structural constraints—notably the difficulty of blinding everyone involved—hamper the conduct of such trials, even if there is room for improvement. Second, the review method itself is a limitation, since the magnitude of effect sizes could not be appropriately determined by a meta-analysis. Third, because apathy was a secondary endpoint in more than half of the included trials, the body of evidence is comparatively less exposed to publication bias, yet selective reporting cannot be ruled out. Fourth, publication bias was not formally assessed, and most reports did not state allocation concealment or pre-randomization concealment explicitly. Fifth, the review was not prospectively registered, which constrains external verification of the planned methods; nonetheless, the search, screening, extraction, and quality appraisal followed a procedure consistent with PRISMA 2020 standards. In addition, several further limitations should be acknowledged: the search was limited in reproducibility and did not include Embase, Scopus, trial registries, or gray literature; only English-language reports were eligible; no proprietary standardized data-extraction form was used; certainty of evidence was not formally appraised using GRADE, a deliberate decision given that the heterogeneity of interventions, comparators, and apathy instruments and the absence of pooled per-outcome estimates precluded a methodologically sound outcome-level GRADE assessment; and earlier drafts included a small number of non-randomized designs, which have now been removed so that the synthesis rests on randomized controlled trials only.

Notably, attrition exceeding 15% was recorded in a considerable proportion of the trials evaluated for quality.

Incorporating newer studies reinforces rather than resolves these methodological constraints on synthesis. Several features of the updated evidence base limit the confidence with which conclusions can be drawn. First, the inability to blind subjects and therapists, an inherent limitation of psychosocial, exercise, and sensory interventions, persists uniformly across the newer trials, so positive findings must be read with the caveat that self-reported BPSD outcomes may be inflated by expectation effects. Second, appraising internal validity is hampered by uncertain allocation concealment in several trials (see Table 2), explicit intention-to-treat analysis in only a minority, and attrition at or near the PEDro threshold in some studies (e.g., ref. [101] reported exactly 85% follow-up at 14 weeks with 12/80 dropouts). In PD, synthesis is further constrained by underpowered trials (e.g., ref. [72], terminated early because of the COVID-19 pandemic) and high rates of protocol violation (e.g., ref. [57], with participants missing more than half of the sessions). Third, and most consequential for comparing apathy outcomes across studies, the primary endpoints were markedly heterogeneous; although some dementia studies did use apathy-specific instruments as primary or co-primary outcomes (see Table 2), this variability limits comparability and underscores the continuing absence of a standardized approach to measuring apathy as the primary outcome in intervention research. Fourth, the included trials combined validated apathy-specific instruments with proxy or broader behavioral measures: 49 of the 62 trials used a validated apathy-specific instrument (e.g., the Apathy Evaluation Scale, the NPI apathy domain, the Apathy Scale, the Lille Apathy Rating Scale, the Apathy Inventory, DAIR, or APADEM-NH), whereas 13 relied on proxy or behavioral outcomes (engagement, passivity, social-withdrawal, mood, negative-symptom, or behavior-observation scales); the per-study classification is provided in Supplementary Materials, Table S3, and conclusions resting on proxy outcomes should be regarded as more tentative and less specific to apathy. Fifth, two included trials used a randomized crossover design (Kolanowski et al. 2005 [22] and Moyle et al. 2013 [23]; flagged in Table 1); because carry-over effects cannot be excluded in progressive disorders, their results were interpreted as within-subject (between-condition) rather than parallel-group comparisons and were weighted cautiously, whereas non-randomized designs (quasi-experimental or stepped-wedge) were excluded. Finally, study-level screening logs were not retained, so individual full-text exclusion decisions cannot be independently verified; exclusions are reported by primary reason in Supplementary Materials, Table S2.

The domain-level risk-of-bias assessment across all 62 included studies, mapped from PEDro items onto Cochrane risk-of-bias domains, revealed the characteristic profile of non-pharmacological intervention research. Random group allocation was universal (100%) and baseline similarity common (72.5%), but concealed allocation was reported in fewer than one-third of trials (27.5%), so selection bias remains a substantive concern. Performance bias was the most pervasive source of risk: subject blinding was reported in only 15.9% and therapist blinding in only 18.8% of studies, reflecting the intrinsic difficulty of masking participants and interventionists to psychosocial, exercise-based, sensory, and technology-assisted treatments. Detection bias was better controlled, with at least one blinded outcome assessor in 63.8% of trials. Attrition was mixed: adequate follow-up (less than 15% dropout) was reported in 68.1%, but intention-to-treat analysis in only 52.2%, suggesting that missing-data handling may have introduced bias in a substantial share of trials. Reporting bias was generally low, with between-group comparisons reported in 89.9% and point estimates with measures of variability in 91.3% of studies. At the aggregate Cochrane domain level, reporting bias showed the highest compliance (90.6%), followed by detection bias (63.8%) and attrition bias (60.1%), while selection bias averaged 66.7% across its three constituent items and performance bias was the weakest domain (17.4%). Overall, 42.0% of studies were rated as high-quality (PEDro ≥ 7), 42.0% moderate (PEDro 5–6), and 15.9% poor (PEDro ≤ 4), with the large majority graded OCEBM Level B (85.5%), followed by Level A (5.8%) and Level C (8.7%). These patterns indicate that, while the evidence base is methodologically acceptable in terms of randomization, outcome reporting, and assessor blinding, the near-universal absence of participant and therapist blinding, a structural limitation of non-pharmacological trials, must temper interpretation of positive findings, particularly self-reported apathy outcomes susceptible to expectation effects.

#### 4.2.2. Relation to Other Reviews

Few reviews specifically target apathy outcomes in aging neurocognitive disorders after NPTs [103,104,105]. We have shown that several NPTs, alone or combined with other treatments, may broaden the options for managing apathy. A meta-analysis is nonetheless needed to compute effect sizes and gauge heterogeneity in context, a need now partly met: Jia et al. [100] synthesized nine RCTs (n = 356) and confirmed that physical exercise significantly reduces apathy in older adults (SMD = −0.32) with low heterogeneity (I^2^ = 14.7%), providing a pooled quantitative estimate of effect in this domain.

Our conclusions converge with the small number of earlier reviews addressing apathy outcomes after non-pharmacological treatment [103,104,105]. Verkaik et al. [103] judged multisensory behavior therapy (Snoezelen) potentially effective; Lane-Brown et al. [104] reported apparent benefit from music therapy and cognitive rehabilitation, albeit not in dementia samples specifically; and Politis et al. [11] argued that a creative activity therapist attuned to a patient’s personal interests can help the apathetic AD patient. In a similar vein, Treusch et al. [48] highlighted physical activation and biography-orientated mobilization as motivating strategies for people with dementia, while Brodaty and Burns [105] concluded that individually delivered therapeutic activities carry the strongest available evidence in dementia. Family education is also important, since apathetic patients are frequently misread as lazy or uncooperative [10]. Mele et al. [98] provided evidence that exercise and mindfulness-based interventions in PD may improve apathy scores, specifically through goal-directed behavior change and social engagement, provided participants retain minimal cognitive impairment. The VR meta-analysis [95] adds a further dimension, suggesting that virtual-reality technologies may reduce apathy alongside aggression and depression, though the evidence base is predominantly quasi-experimental and methodologically heterogeneous.

### 4.3. Implications for Future Research

#### 4.3.1. Methodology and Safety

NPTs appear to be well-tolerated and are relatively safer than pharmacological ones. Expert consensus suggests that NPTs are useful to consider as therapy for people presenting with different neurocognitive and psychiatric diseases at all stages, with evidence of apathy across behavioral, cognitive, and emotional domains [106]. However, the design of studies assessing non-pharmacological interventions is inherently less robust, often relying on single blinding. Due to heterogeneity of the sample populations and the syndromic nature of apathy, its symptoms rather form clusters within different NPI symptoms which are consistent across studies defining potential sub-syndromes [107]. In this context, initiatives and consortia on apathy are active and recommendations [108,109] on the design of clinical trials on apathy have recently been published. Endorsement of the CONsolidated Standards of Reporting Trials (CONSORT) statement can substantially improve the completeness and transparency of these trials (e.g., ref. [67]). Future research should also systematically report concurrent medications, as it is often unclear if participants are taking drugs that might induce or alleviate apathy [22,35].

#### 4.3.2. Refining Parkinson’s Disease Research

While research on apathy treatment in Alzheimer’s dementia is ongoing, work in Parkinson’s disease (PD), where apathy is highly prevalent, remains limited but is growing. Newer PD evidence includes mindfulness training [72], dance interventions [57,73,75], high-frequency repetitive transcranial magnetic stimulation (rTMS) [76], and aerobic cycling [81]. To date, only rTMS has shown robust apathy reduction (*p* < 0.0001), while mindfulness and dance primarily prevented worsening of symptoms. Effective PD interventions appear to target goal-directed behavior through social engagement, although cognitive impairment may limit treatment responsiveness [98]. Adequately powered confirmatory trials that facilitate symptom expression and engagement, with apathy as a primary outcome, are warranted in the PD population.

#### 4.3.3. Standardization of Assessment and Diagnostic Criteria

The lack of established tools for apathy contributes to heterogeneity and complicates the appraisal of outcomes. Notably, most studies assess apathy as a secondary measure within the broader NPI, which lacks sufficient sensitivity. Future trials may incorporate the 2018 Diagnostic Criteria for Apathy (DCA) or the 2021 Consensus Criteria to reduce participant heterogeneity [16,18]. For assessment, the DAIR and the Apathy Evaluation Scale (AES) are among the highest quality tools for Alzheimer’s Disease [110]. Emerging research also highlights the Mild Behavioral Impairment Checklist (MBI-C) as a vital case-ascertainment instrument for detecting emergent apathy in pre-dementia states, such as MCI [111]. Researchers are encouraged to use multidimensional scales (e.g., Apathy Motivation Index or Dimensional Apathy Scale) to capture differential effects across apathy subtypes [110].

#### 4.3.4. Personalized Therapy

Generic approaches to activity may often fail and clinical practice should move toward “tailor-made” approaches that align with a patient’s lifelong roles, personal interests, and preserved abilities [106]. A critical component in this line is the inclusion of sensory preferences (visual, auditory, olfactory, tactile, and gustatory), which should be systematically collected via structured interviews or observation by exposure [106]. Future research should focus on developing standardized tools to capture these aspects reliably.

#### 4.3.5. Technology-Based Interventions

The emergence of technology-based interventions, including immersive and group-based VR [53,96], serious exergames [66], and tablet-based activation [78], marks a new frontier. Information and communication technologies (ICT) offer notable strengths, including increased ecological validity and the capacity to record objective, longitudinal data on treatment adherence and “indirect” indicators such as voice and movement [106]. While early results are promising, most evidence remains at the pilot or feasibility stage, as shown in the VR meta-analysis [95]. Future efforts should prioritize adequately powered RCTs of ICT interventions with validated apathy-specific primary outcomes and investigate “closed-loop cognition,” in which algorithms adapt intervention difficulty in real time to patient performance [106].

#### 4.3.6. Combination Strategies

Future trials should investigate combination treatment strategies, such as physical exercise paired with mindfulness or ICT-based tools combined with caregiver training. Similarly, treatment development should also examine the interaction between NPTs and pharmacological agents, such as using a non-pharmacological lead-in phase [105]. Investigating interventions in different care contexts is essential, as strategies effective at home may not be optimal for long-term care environments. Caregivers remain critical to treatment success, as they can increase daily functioning by prompting patients to initiate activities, using visual cues, and establishing routines [10,106,112].

#### 4.3.7. Prevention and Disease Progression

Beyond direct interventions, because apathy is a significant risk factor for the conversion from mild cognitive impairment to dementia, early intervention in MCI or subjective cognitive decline may offer opportunities to alter the trajectory of neurodegeneration [109]. Future research should also investigate the cost-effectiveness and long-term sustainability of interventions [106]. Speculation regarding dietary or metabolic modification is beyond the scope of the present review, which is confined to randomized non-pharmacological intervention trials.

## 5. Conclusions

This systematic review of randomized controlled trials found that a range of non-pharmacological interventions—including psychosocial, exercise-based, creative, technology-assisted, and brain stimulation approaches—may reduce apathy in older adults with neurocognitive disorders. Although no single intervention class was clearly superior, effects were frequently short-lived, and the overall certainty of evidence is limited. The evidence base is dominated by Alzheimer’s disease and Parkinson’s disease, while data for Huntington’s disease and mild cognitive impairment remain insufficient to support disease-specific conclusions. Notably, fewer than half of the included trials (30/62, 48%) demonstrated a statistically significant between-group benefit for apathy, and apathy was a primary outcome in only a subset, so the evidence should be read as encouraging but preliminary rather than confirmatory.

Given the persistent methodological limitations—small sample sizes, the inability to blind participants and therapists in most designs, inconsistent use of validated apathy-specific outcome measures, and predominantly short follow-ups—current findings should be interpreted cautiously and do not yet justify clinical recommendations. Future trials should adopt apathy as the primary endpoint, use standardized apathy-specific instruments, report effect sizes with precision estimates, employ sham- or active-controlled designs where feasible, and include longer follow-ups to establish the durability of effects.

## Figures and Tables

**Table 1:** Randomized controlled trials on non-pharmacological interventions in individuals with apathy in neurocognitive disorders. The Outcome column reports, for each trial, the direction and statistical significance of the effect on apathy, whether it was a between-group or within-group effect, the timing or durability of the effect where reported, and whether apathy was a primary or secondary measure. In the “Apathy measure (Primary measure)” column, the first entry is the apathy instrument on which study eligibility was based on and the parenthetical entry is the study’s primary outcome measure(s).

Trial	*N* (exp./con.)	Mean Age (Years)	Intervention	Context	Treatment Duration (Weeks)	Apathy Measure (Primary Measure)	Outcome	Comments
Baker et al. (2001) [24]	33 (15/18)	78	MSS	Day care centers	4	INTERACT Short/BRS/BMD (INTERACT Short/BRS/BMD)	Significant reduction—between-group; apathy primary. BRS social disturbance MD 0.84, 95% CI [−1.59, −0.09], *p* = 0.029.	- 7 VaD and 10 mixed dementia patients included. - Benefit declined following end of intervention
Cott et al. (2002) [25]	86 [ITT]	82	Tailored conversation while walking in pairs (walk-and-talk group), or conversation while sitting in pairs (talk-only group), or neither of the two	Long-term care facilities	16	Engagement, communication (FACS, LPRS)	No significant effect—between-group; apathy secondary. Social-communication post-test change *p* > 0.05.	AD dementia subjects
Schrijnemaekers et al. (2002) [26]	151 (15 male)	85	Emotion-oriented care vs. usual care	NH	48 (1 year)	Dutch Behavior Observation Scale for Psychogeriatric Inpatients (GIP)	No significant effect—between-group; apathy secondary.	
Baker et al. (2003) [27]	136 (65/71)	81 (MSS group) 83 (activity group)	MSS	Day Hospital (UK), Psycho-geriatric wards (the Netherlands, Sweden)	4	BRS/BMD/Gedragsobservatieschaal voor de Intramurale Psychogeriatrie—GIP/INTERACT Short (BRS/BMD/GIP/INTERACT Short)	No significant effect (subgroup benefit only)—between-group; apathy primary. MMSE MD −0.3, 95% CI [−1.4, 0.7].	- 3 country sites
Politis et al. (2004) [28]	36 (18/18)	84.4 (‘kit’ group) 83.5 (one-on-one group)	Kit-based activity intervention	Model care facility for patients with dementia	4	NPI (NPI)	No significant effect—between-group; apathy primary. NPI apathy Z = −0.526, *p* = 0.60.	
Chapman et al. (2004) [29]	54 (26/28)	76.38	Cognitive communication stimulation (Donepezil-plus) vs. Donepezil-only	At-home dementia patients and their caregivers	48	NPI (NPI)	No significant effect—between-group; apathy primary. Apathy SI MD −1.10, 95% CI [−2.33, 0.13].	- Differences in change scores were at *p* = 0.0773 for the apathy severity index, *p* = 0.0556 for a group factor, *p* = 0.0618 for a Group × Time factor. - Effect size was 0.45, in apathy severity index
Lai et al. (2004) [30]	101 [ITT]	85.6	Specific reminiscence/discussion of the patient’s life history individually based on Hellen’s [31] “LSB’’ concepts, or discussion on other themes, or no intervention	NHs	6	Social engagement and well-being (SES, WIB)	No significant effect—between-group; apathy secondary. SES effect size 0.374.	DSM-IV dementia, moderate and severe.
Finnema et al. (2005) [32]	146	83.8 (treatment group) 83.6 (control group)	Integrated emotion-oriented and usual care or usual care alone	NHs	36	BIP (BIP, CSDD, C-MAI, GRGS, PGCMS)	Worsening of apathy (marginal)—between-group; apathy primary. Apathetic behavior d = 0.06.	Moderate and severe AD subjects
Holmes et al. (2006) [33]	32	84.9	Live interactive music	NHs	Immediately	DCM (DCM)	Significant increase in engagement—between-condition; apathy primary. Median engagement live (+1) vs. recorded/silent (0).	Only immediate effects of a 30 min intervention are reported.
Staal et al. (2007) [34]	24 (12/12)	80.33 (experimental group) 72 (control group)	Multisensory behavior therapy	Geriatric psychiatric unit	Immediately	Assessment of negative symptoms in Alzheimer’s disease scale (assessment of negative symptoms in Alzheimer’s disease scale)	Significant reduction—between-group; apathy primary. MSBT 3.15 vs. Cg −2.31, *p* = 0.04.	Effects after six sessions of a 25–30 min intervention are reported.
Tadaka and Kanagawa (2007) [35]	24 AD (12/12) and 36 VD (18/18)	83.29	Reminiscence	Geriatric health services facility in Japan	24	MOSES (MOSES)	Significant reduction in social withdrawal—between-group; apathy primary. Withdrawal Iv 16.8 vs. Cg 19.5, *p* = 0.059 (ANCOVA).	- Vascular dementia patients included - Apathy non-specific outcome reported
Gitlin et al. (2008) [36]	60 (30/30)	79	Tailored activity program or wait-list control	At-home dementia patients and their caregivers	16	Activity engagement measured using a 5-item, investigator-developed index of caregiver report of patient in the past two weeks	Significant improvement—between-group; apathy primary. Activity engagement Cohen’s d = 0.61, *p* = 0.029.	Apathy non-specific outcome reported
Raglio et al. (2008) [37]	59	84.4 (treatment group) 85.8 (control group)	Music therapy or educational/entertainment activities	NH	16	NPI	Significant improvement—between-group; apathy primary. Global NPI Cohen’s d = 1.04.	- Moderate and severe AD, VD and mixed dementia cases. - Effect sizes only for NPI global score changes
Tappen and Williams (2009) [38]	36 (3 men)	83.8 (treatment group) 90.26 (control group)	Therapeutic conversation or care as usual	NH	64	AD-RD mood scale	Significant reduction—between-group; apathy secondary. Treatment group showed significantly less apathy than control.	- Moderate and severe AD dementia cases.
Hsieh et al. (2010) [39]	61	77.56	Reminiscence group therapy	NH	12 on average	AES-C (AES-C, NPI, GDS)	Significant reduction (behavior/cognition)—within-group; apathy primary. Apathy (behavior) Z = −3.10, *p* = 0.002.	- Not double-blind - Greater baseline emotional apathy in the experimental group (*p* = 0.04) - DSM-IV criteria for dementia
Lam et al. (2010) [40]	74 (37/37)	83.45	Individualized daily activities (functional enhancement program)	Social centers and old -aged home for the elderly in Hong Kong	16	NPI (NPI)	No significant effect—between-group; apathy secondary. Within-group *p* = 0.04 but between-group *p* > 0.05.	
Niu et al. (2010) [41]	32 (16/16)	80.56 (experimental group) 79.13 (control group)	Cognitive stimulation	A military sanatorium in China	10	NPI (NPI)	Significant reduction—between-group; apathy secondary. CST change −1.06 (0.85) vs. Cg −0.31 (0.60), *p* = 0.017.	
Raglio et al. (2010) [42]	60 (30/30)	85.4 (experimental group) 84.6 (control group)	Music therapy	NHs	24	NPI (NPI)	Significant reduction—between-group; apathy primary. Experimental T0–T2 change *p* < 0.001.	
Ferrero-Arias et al. (2011) [43]	146 (73/71)	83.6	Music and art therapy and psychomotor activity or free activities in the day room	NHs or daycare centers	8	NPI/DAIR (NPI/DAIR)	Significant reduction—between-condition (crossover); apathy primary. MD 0.21, 95% CI [0.07, 0.34], *p* < 0.005.	Institutionalized or daycare dementia patients
Hattori et al. (2011) [44]	39 (20/19)	75.3 (experimental group) 73.3 (control group)	Art therapy	Outpatient clinic of a clinical center	12	Apathy Scale (Apathy Scale)	No significant effect—between-group; apathy secondary. Intergroup comparison *p* = 0.090.	
Kolanowski et al. (2011) [45]	128	86	Activities tailored to functional level (FL), and personality style of interest (PSI) alone or in combination (FL + PSI).	NHs	3	Passivity and engagement (PDS, C-MAI, time on task, intensity of engagement, ARS, DMPT)	Significant improvement in engagement only—within-group; apathy secondary. Passivity interaction *p* = 0.23.	- Moderate and severe dementia - MMSE_PSI_ > MMSE_PSI+FL_ at baseline - Education years in PSI > Education years in FL, PSI + FL and control groups _FL_ at baseline.
Kolanowski et al. (2005) [22]	30 (crossover)	82.3	NDB-derived activities (skill + interest matched)	4 nursing homes, USA	12 days per condition	Passivity in Dementia Scale (PDS)	Significant reduction in passivity vs. baseline—within-group; apathy primary. Passivity change vs. baseline (mean −3.10, 95% CI [−4.4, −1.8]).	NDB activities significantly more effective for passivity than skill-only matching
Maci et al. (2012) [46]	14 (7/7)	75 (treatment group) 70.3 (control group)	Cognitive stimulation, physical activity, and socialization	Clinical dementia center in Spain	12	AES (AES)	Significant reduction—between-group; apathy primary. Treatment group 60.6 → 51.0, *p* < 0.05.	
Moyle et al. (2013) [23]	18	85.3	PARO vs. interactive reading	Residential care facility	10	AES (QOL-AD, RAID, AES, GDS, Revised Algase Wandering Scale—NH version, OERS)	No clinically significant effect—between-group; apathy secondary. Cohen’s d = 0.2 (small).	- Crossover design - Mid- to late-stage or DSM-IV-TR criteria for probable dementia
Telenius et al. (2015) [47]	163 (82/81)	86.9 (experimental group) 86.4 (control group)	Individually fitted, average 18, 50–60 min sessions of high-intensity physical exercise or control activity	18 NHs	12	NPI-Q-Apathy independently (BBS, NPI-Q, BI, CSDD)	Significant reduction—between-group; apathy primary. Apathy change d = 0.3, *p* = 0.048.	Dementia diagnoses not specified (CDR score 1 or 2)
Treusch et al. (2015) [48]	117 (67/50)	80.12	“Biography-orientated mobilization”	18 NHs in Berlin	40	AES/NPI (AES/NPI)	Preventive effect (control worsened)—between-group; apathy primary. Treatment difference t = 2.63, *p* = 0.01.	
Valenti Soler et al. (2015) [49]	101 (at NH) to 110 (at day care center)	84.7	NAO, PARO (phase 1) vs. PARO, real dog (phase 2) vs. control	Parallel NH and daycare center	24	APADEM-NH/AI (G.D.S., severe MMSE, MMSE, NPI, APADEM-NH, AI, QUALID)	Significant improvement (cognitive inertia/NPI apathy)—between-group; apathy primary. APADEM-NH total −3.55 (*p* < 0.05).	- Randomization carried out only in NHs—NAO followed by PARO was implemented in the daycare center. - 84–88% AD, 7–11% mixed dementia, 1–2% DLB, 1–3% PDD or FTD. - 88.5% (phase 1) and 90% (phase 2) were women.
Amieva et al. (2016) [50]	653 (499/154) cognitive training N = 170 reminiscence therapy N = 172 individualized cognitive rehabilitation program N = 157	78.7	Individual cognitive therapies cognitive training (group sessions), reminiscence therapy (group sessions), individualized cognitive rehabilitation program (individual sessions) vs. controls	40 French clinical sites	104	NPI/Apathy Inventory (rate of survival at two years for patients without moderately severe to severe dementia)	No significant effect—between-group; apathy secondary. Individual rehabilitation vs. usual care at 24 months *p* = 0.9656.	Apathy secondary measure
Di Domenico et al. (2016) [51]	32	70.46 (AD patients) 70.88 (control group)	Mixed design with a 2 (Conditioned Stimulus-CS: Non-word vs. Activity) within-subject × 2 (Unconditioned Stimulus-US: Neutral vs. Positive) × 2 (Group: AD patients vs. Healthy Subjects) between-subjects manipulation	Outpatient clinics	12	AES (AES)	Significant improvement in motivation—within-group; apathy primary. CS-activity “wanting” ηp^2^ = 0.551 (large).	- Probable AD (NINCDS-ADRDA criteria) - AES > 45, GDS < 20 included - AD with apathy vs. healthy older subjects
Ikemata and Momose (2017) [52]	37	86.89 (treatment group) 86.74 (control group)	Progressive muscle relaxation by seven groups of muscles: forearm and upper arm; lower leg and front thigh; lower leg and rear thigh; chest; shoulder; forehead; periorbital and lower law	NH	13	NPI-NH (NPI-NH, NM scale)	Significant reduction (interest/volition)—within-group; apathy primary. NM-scale analysis *p* < 0.05.	- Lack a diagnosis based on clinical criteria. - Clinical type of dementia not specified in 27 subjects.
Manera et al. (2016) [53]	57	75 (MCI patients) 76.3 (AD patients)	Attentional task (written condition vs. virtual reality condition)	Memory center and research unit	N/A	AI	No significant effect—between-group; apathy primary. Diagnosis interaction F(1,55) = 0.51, *p* = 0.480.	- Mild to moderate dementia (ICD-10 criteria) and MCI (NIA-AA criteria) subjects were compared - Exploratory study
Rajkumar et al. (2016) [54]	273	85.7 (with apathy 84.7)	Evidence-based person-centered care (control) or additional NICE/Alzheimer’s Society/Department of Health-guided antipsychotic review (AR) alone, or in combination with either 1 h/w exercise (EX) or 1 h/w social interaction (SI) (or 20% increase if existing at baseline)	Nursing home (NH)	36	NPI-NH (Antipsychotic reduction rate. NPI-NH)	Small between-group effect—between-group; apathy primary. EX-arm apathy change −1.05 (4.13).	- Strong design - Clinical diagnostic criteria for type of dementia not stated - Apathy at baseline associated with study withdrawal (χ^2^ = 8.04; df = 1; *p* = 0.005) - About 30% completed the study
Sánchez et al. (2016) [55]	32 (11/10) N = 11 for one-to-one activity session	85.4	Multisensory stimulation environment vs. one-to-one activity session vs. control group	Specialized dementia elderly center	16- and 8-week follow-up	NPI (NPI)	Significant reduction in global NPS—between-group; apathy secondary. NPI η^2^ = 0.238 (large).	- Improvements found during the intervention were lost in the follow-up period
Cugusi et al. (2015) [56]	20 PD patients (10/10)	67.3 ± 7.8	Nordic walking program (NW)	Movements disorder center—outpatients	12	Short version of the Starkstein Apathy Scale	Significant reduction—between-group; apathy secondary. SAS: NWg 22.8 → 16.5 vs. Cg 22.6 → 23.6; across-group *p* < 0.0005.	
Hashimoto et al. (2015) [57]	46 PD patients (15 dance group/ 17 PD exercise group/ 14 control group)	67.9 ± 7.0 (dance group) 62.7 ± 14.9 (exercise group) 69.7 ± 4.0 (control)	Dance group	PD patient associations	12	AES (AES)	Significant reduction—between-group; apathy secondary. Group × Time interaction on AS F(2,42) = 8.0, *p* < 0.05; η^2^ = 0.26 (large); dance 14.7 → 10.2. Quasi-randomized (cluster) design.	- Men-to-women ratio not equal in each group
King et al. (2015) [58]	58 PD patients (home group 17/ individual group 21/ class group 20)	63.9 ± 8	Physical exercise	Movement disorders clinic	6	Lille Apathy Rating Scale (LARS)	No significant effect—between-group; apathy secondary. Home-group MD −0.40, 95% CI [−3.3, 0.04].	- Lack of a non-exercising control group - Only 4 weeks of exercise -No follow-up period
Berardelli et al. (2018) [59]	20 PD patients with a diagnosis of psychiatric disorder (9/9)	CBT: 60.5 ± 5.6 Psychoeducational Group: 57.1 ± 5.3	12-week cognitive behavioral therapy (CBT) group or a psychoeducational protocol	Outpatient clinic for movement disorders	12	AES	Significant reduction—between-group; apathy secondary. CBT improved AES/NMS significantly vs. control.	- Small sample size
Friedmann et al. (2015) [60]	40 assisted living residents with cognitive impairment (22/18)	80.72 ±9.12	Pet-assisted living intervention (PAL) (n = 22) or reminiscing (n = 18) twice/week for 12 weeks	Residences that are part of a network of small family style	12	Zimmerman’s short version of the AES	No significant effect (within-group trend only)—within-group; apathy primary. Apathy interaction ES 0.12.	- Only people comfortable with animals are appropriate participants in animal-assisted intervention or activity - Study was limited due to limited number and type of facilities included and lack of specific dementia diagnosis
Balzotti et al. (2019) [61]	30 (20/10)	GVT group: 82.4 ± 5.7	Two intervention programs, the gesture-verbal treatment (GVT) and the doll therapy (DT). The control group participated only in standard rehabilitative therapies	NH	12	NPI	Significant reduction—between-group; apathy primary. Between-group difference on apathy change *p* = 0.0002.	- Sample size was small - Psychosocial and caregiver operators may have referred patients to the study whom they believed might particularly profit from such approaches
Tang et al. (2018) [62]	77 (37/39)	75.88 ± 5.09	Music intervention program vs. control group	NH	12	AES	Significant reduction—between-group; apathy primary. AES change Z = 4.516, *p* < 0.001.	-it was not examined whether intervention effects can last after 12 weeks or whether there is greater improvement with a >12 weeks intervention duration
Schall et al. (2018) [63]	44 people with dementia: 32 had AD (72.7%), seven vascular dementia (15.9%), two PD dementia (4.6%) and three dementia of unclear etiology (6.8%). (25/19)	Intervention Group: 75.1 ± 7.70 Control: 76.4 ± 8.68	The intervention consisted of six different guided art tours (60 min), followed by art-making in the studio (60 min). Independent museum visits served as a control condition	People with mild to moderate dementia and their informal caregivers living in Frankfurt am Main and the Rhine-Main area.	6 sessions	NPI	Significant reduction—between-group; apathy secondary. NPI apathy 12.4 → 9.27, t = 2.52, *p* = 0.025.	- Self-report of some outcomes may have been a limitation -Examined sample only partially met the requirements of a random sample
İnel Manav and Simsek (2019) [64]	32 (16/16)	74.44 ± 4.48	Reminiscence therapy that was supported with internet-based	NH	12	Apathy Rating Scale (ARS)	Significant improvement (post-test)—between-group; apathy primary. Intervention MD 8.63 (4.95), *p* = 0.001.	- Relatively small sample size -This study is randomized and controlled but not single-blind
Trinkler et al. (2019) [65]	19 patients with HD (8/11)	The patients: 43–78 years with median 53 years Control group: 44–72 with median 53 years	A lyrical dance form, practiced for two hours per week over five months	Patients recruited at the genetics department of the Pitié-Salpêtrière University Hospital	20	LARS	No significant difference—between-group; apathy primary. Z = −0.32, *p* = 0.75.	- This study included only patients with a relatively high functional score - Relatively small sample size
Robert et al. (2021) [66]	91 (37/54)	Mean age = 81.7 years	Serious exergame	Outpatients consulting memory centers, daycare centers and NH	24	NPI and Apathy Inventory	Significant reduction (preventive)—between-group; apathy primary. Time × Group interaction *p* = 0.032.	- The number of subjects was relatively small, and their profiles were quite heterogeneous - Study performed in different contexts, patients had different cognitive and motor profiles and did not benefit from the same amount of physical and cognitive stimulation
Yang et al. (2021) [67]	32 (16/16)	84.5 ± 9.5	Horticultural therapy	Dementia care unit	12	AES-I	Significant reduction at T1—between-group; apathy primary. Z = −2.685, *p* = 0.007.	- Sample size was relatively small - Apathy was assessed by asking the informants - The severity of dementia may affect the effects of HT - 3 participants attended the make-up session alone, which might have led to the absence of group effect
Padala et al. (2020) [68]	9/11	77.3	rTMS (left DLPFC)	Geriatric center, USA	2 weeks (6 sessions)	AES-C	Significant improvement at 4 weeks—between-group; apathy primary. MD = −10.1, 95% CI [−15.9, −4.3], *p* = 0.002.	- Double-blind pilot; effects not sustained at 8/12 weeks
Zhuo et al. (2025) [69]	(78) 39/39; AD (55.13%), VD (30.77%), or mixed dementia (12.82%)	85.29	Creative expressive art-based storytelling (CrEAS-AC)	Geriatric wards (tertiary hospital), China	12	AES-I (Apathy Evaluation Scale-Informant)	Significant reduction at 12 and 24 weeks (sustained)—between-group; apathy primary. MD = −1.90, 95% CI [−2.53, −1.27], *p* < 0.001; Cohen’s d = −0.67.	- Single-blind - Effects maintained for 3 months post-intervention - Improved QoL.
Li et al. (2025) [70]	(80) 40/40 (dementia of any type and severity; 53.8% in the mild stage at baseline)	83.23	Home-based aromatherapy (lavender oil inhalation)	Homes, Hong Kong	3	Chinese NPI (CNPI)—apathy domain	No significant effect—between-group; apathy secondary. β = 0.941, 95% CI [−0.874, 2.755], *p* = 0.310.	- Waitlist RCT - Significant improvement only in disinhibition and irritability
Yang et al. (2025) [71]	(207) 102/105 (mild-stage dementia-72% with a CDR score of 0.5)	~79	Video-based MTM (exercise + art/painting)	Community service centers, Taiwan	16	NPI-Q—apathy domain	No significant effect—between-group; apathy secondary. Mean change −0.1 (0.8) vs. −0.1 (0.8), *p*-interaction = 0.638.	- Cluster RCT - Low dropout (1.9%) - Significant only for appetite/eating distress
Buchwitz et al. (2021) [72]	(30) 14/16 PD non-demented (PANDA score ≥ 15)	64.5	IPSUM mindfulness training	Hospital, Germany	8	AES (AES)	Preventive effect (control worsened)—between-group; apathy secondary. Control worsened *p* = 0.01; training stable *p* = 0.360.	- Low power due to COVID-19 termination
Rios Romenets et al. (2015) [73]	(33) 18/15 PD non-demented	71	Argentine tango	Movement clinic, Canada	12	AES (AES)	No significant effect—between-group; apathy secondary (exploratory). p-interaction = 0.904.	- Significantly higher enjoyment and satisfaction
Sajatovic et al. (2017) [74]	(30) 15/15 PD with comorbid depression	70	Group exercise + chronic disease self-management	Fitness center, USA	24	AES (MADRS)	No significant effect—between-group; apathy secondary (depression was primary). Week-12 difference *p* = 0.662.	- Significant improvement in depression
Solla et al. (2019) [75]	(20) 10/10 PD	67.4	Sardinian folk dance	Outpatient clinic, Italy	12	SAS (functional and gait performance)	Preventive effect (control worsened)—between-group; apathy primary. Hedges g = 1.24 (large); interaction *p* = 0.016.	- Pilot study - Large effect sizes for motor performance
Wei et al. (2021) [76]	(50) 25/25 PD -comparison between the apathetic and non-apathetic patients	64	HF-rTMS (right dorsolateral prefrontal cortex)	Hospital ward, China	<1	SAS (SAS)	Significant reduction after rTMS vs. sham—between-group; apathy primary. *p* = 0.005 vs. sham.	- Included EEG/ERP analysis
Yang et al. (2024) [77]	(80) 40/40 AD	72.8	Multi-sensory stimulation	Hospital, China	4	AES	Significant reduction—between-group; apathy primary. Post-test Iv 35.58 vs. Cg 46.8, *p* < 0.001.	
O’Sullivan et al. (2022) [78]	(162) 80/82 dementia general	83.2	Tablet-based activation	Nursing homes, Germany	8	AES-I	No significant effect—between-group; apathy primary.	- Imputed data for 17%
Oliveira et al. (2021) [79]	(54) 28/26 dementia various types	77.4	Tailored activity (TAP)	Outpatient, Brazil	4	NPI-C (apathy)	Significant reduction (pre–post)—within-group; apathy primary. NPI-C apathy change ES 0.4, *p* = 0.007.	- Effective for caregiver burden
Santagata et al. (2021) [80]	(54) 26/28 dementia general	84.1	Doll therapy	Nursing home, Italy	12	NPI (BPSD)	Significant reduction in overall BPSD—between-group; apathy secondary. Grützner scale improvement *p* < 0.0001.	- Reduced delirium incidence
Sacheli et al. (2019) [81]	(35) 20/15 PD	66.8	Aerobic exercise (cycling)	Lab/clinic, Canada	12	AES	No significant effect—between-group; apathy secondary (motor function was primary). Reported as not significant.	
Suemoto et al. (2014) [82]	(40) 20/20 AD	76.5	tDCS (prefrontal)	Hospital, Brazil	4	AES	No significant effect—between-group; apathy primary. Repeated-measures *p* = 0.552.	- Safe but lack of efficacy
Verkaik et al. (2019) [83]	(49) 25/24 dementia general	83.3	Beach Room Stimulation	Nursing home, Netherlands	6	AES	No significant effect—between-group; apathy primary. Chi-square linear trend *p* = 0.91.	- Café control condition was more effective
Vitale et al. (2024) [84]	(28) 13/15 PD	66.1	Biodanza™ (Rolando Toro’s Biodanza)	Rehabilitation, Italy	12	AES	Non-significant Time × Group interaction on apathy (AES F(1,26) = 3.371, *p* = 0.078, η^2^p = 0.115)—between-group; apathy primary. Post hoc comparisons showed the control group worsened (corrected-*p* = 0.039) and scored higher than the Biodanza group at T1 (corrected-*p* = 0.025), while the intervention group remained stable; this preventive pattern derives from post hoc tests and should be interpreted cautiously given the non-significant interaction.	- Behavioral stability maintained

**Table 2:** Methodological quality (PEDro) and level of evidence (OCEBM) of the included randomized controlled trials.

Study	PEDro	PEDro	PEDro	PEDro	PEDro	PEDro	PEDro	PEDro	PEDro	PEDro	PEDro	PEDro	OCEBM
	Random Group Allocation	Allocation Concealed	Baseline Group Similarity	Blinding of All Subjects	Blinding of All Therapists	Blinding of All Assessors of At Least one Key Outcome	Less Than 15% Dropouts	Intention to Treat Analysis of at Least One Key Outcome	Between-Group Statistical Comparisons Reported for at Least One Key Outcome	Point Measurements and Measurements of Variability Provided for at least One Key Outcome	Total Yes	Quality	(Grade of Recommendation)
AD/MCI/Dementia General													
Baker et al. (2001) [24]	Y	N	N	N	N	N	Y	Y	Y	Y	5	Moderate	B
Cott et al. (2002) [25]	Y	Y	Y	N	N	Y	Y	Y	Y	Y	8	High	B
Schrijnemaekers et al. (2002) [26]	Y	N	Y	N	N	N	Y	Y	Y	Y	6	Moderate	B
Baker et al. (2003) [27]	Y	N	N	N	N	N	Y	Y	Y	Y	6	Moderate	B
Politis et al. (2004) [28]	Y	N	N	N	N	Y	Y	Y	Y	Y	6	Moderate	B
Chapman et al. (2004) [29]	Y	Y	N	N	N	Y	N	Y	Y	Y	6	Moderate	B
Lai et al. (2004) [30]	Y	Y	Y	N	Y	Y	Y	Y	Y	Y	9	High	B
Finnema et al. (2005) [32]	Y	N	Y	N	Y	Y	N	N	N	Y	5	Moderate	B
Holmes et al. (2006) [33]	Y	N	N	Y	Y	Y	Y	N	N	N	5	Moderate	B
Staal et al. (2007) [34]	Y	N	Y	N	N	Y	Y	N	Y	Y	6	Moderate	B
Tadaka and Kanagawa (2007) [35]	Y	Y	N	N	Y	Y	N	Y	N	N	5	Moderate	B
Gitlin et al. (2008) [36]	Y	Y	Y	N	Y	Y	Y	Y	N	N	7	High	B
Raglio et al. (2008) [37]	Y	N	Y	N	N	Y	Y	N	Y	Y	6	Moderate	B
Tappen and Williams (2009) [38]	Y	N	N	N	N	Y	N	N	Y	Y	4	Poor	C
Hsieh et al. (2010) [39]	Y	N	Y	N	N	N	Y	N	N	Y	4	Poor	C
Lam et al. (2010) [40]	Y	N	Y	Y	Y	Y	N	Y	Y	Y	8	High	B
Niu et al. (2010) [41]	Y	N	Y	N	N	Y	Y	Y	Y	Y	7	High	B
Raglio et al. (2010) [42]	Y	N	N	N	N	Y	Y	N	Y	Y	5	Moderate	B
Ferrero-Arias et al. (2011) [43]	Y	Y	Y	N	N	Y	N	Y	Y	Y	7	High	B
Hattori et al. (2011) [44]	Y	N	Y	N	N	N	Y	N	Y	Y	5	Moderate	B
Kolanowski et al. (2011) [45]	Y	Y	Y	N	N	Y	Y	Y	Y	Y	8	High	B
Maci et al. (2012) [46]	Y	N	N	N	N	Y	N	N	Y	Y	4	Poor	C
Moyle et al. (2013) [23]	Y	N	Y	N	N	Y	N	N	Y	Y	5	Moderate	B
Kolanowski (2005) [22]	Y	N	Y	N	N	Y	Y	N	Y	Y	6	Moderate	B
Telenius et al. (2015) [47]	Y	Y	Y	N	N	Y	Y	Y	Y	Y	8	High	B
Treusch et al. (2015) [48]	Y	N	Y	N	N	Y	Y	Y	Y	Y	7	High	B
Valenti Soler et al. (2015) [49]	Y	N	Y	N	Y	Y	Y	N	Y	Y	7	High	B
Amieva et al. (2016) [50]	Y	Y	Y	N	N	Y	Y	Y	Y	Y	8	High	B
Di Domenico et al. (2016) [51]	Y	N	N	Y	N	N	N	N	Y	Y	4	Poor	C
Ikemata and Momose (2017) [52]	Y	N	Y	N	N	N	Y	N	Y	Y	5	Moderate	B
Manera et al. (2016) [53]	Y	N	N	N	N	N	N	Y	Y	Y	4	Poor	C
Rajkumar et al. (2016) [54]	Y	Y	Y	N	Y	Y	N	N	Y	Y	7	High	B
Sánchez et al. (2016) [55]	Y	N	Y	N	N	N	Y	N	N	N	3	Poor	C
Friedmann et al. (2015) [60]	Y	N	Y	N	N	N	Y	N	Y	Y	5	Moderate	B
Balzotti et al. (2019) [61]	Y	N	Y	N	N	Y	Y	N	Y	Y	6	Moderate	B
Tang et al. (2018) [62]	Y	N	Y	N	N	N	Y	Y	Y	Y	6	Moderate	B
Schall et al. (2018) [63]	Y	N	Y	N	N	N	Y	N	Y	Y	5	Moderate	B
İnel Manav, Simsek (2019) [64]	Y	N	N	N	N	N	Y	Y	Y	Y	6	Moderate	B
Robert et al. (2021) [66]	Y	N	Y	N	N	Y	Y	N	Y	Y	6	Moderate	B
Yang et al. (2021) [67]	Y	Y	Y	N	N	Y	Y	Y	Y	Y	8	High	B
Padala et al. (2020) [68]	Y	Y	N	Y	Y	Y	Y	N	Y	Y	8	High	B
Zhuo et al. (2025) [69]	Y	Y	Y	N	N	Y	Y	Y	Y	Y	8	High	A
Li et al. (2025) [70]	Y	Y	Y	N	N	Y	Y	Y	Y	Y	8	High	A
Yang et al. (2025) [71]	Y	N	N	N	N	N	Y	N	Y	Y	4	Poor	B
Yang et al. (2024) [77]	Y	N	Y	N	N	N	Y	Y	Y	Y	6	Moderate	B
O’Sullivan et al. (2022) [78]	Y	Y	Y	N	N	Y	N	Y	Y	Y	7	High	A
Oliveira et al. (2021) [79]	Y	Y	Y	N	N	N	Y	Y	Y	Y	7	High	B
Santagata et al. (2021) [80]	Y	N	Y	N	N	N	Y	Y	Y	Y	6	Moderate	B
Suemoto et al. (2014) [82]	Y	N	Y	Y	Y	Y	Y	Y	Y	Y	9	High	B
Verkaik et al. (2019) [83]	Y	N	Y	N	N	Y	N	Y	Y	Y	6	Moderate	B
**Parkinson’s Disease**													
Cugusi et al. (2015) [56]	Y	N	Y	N	N	N	Y	N	Y	Y	5	Moderate	B
Hashimoto et al. (2015) [57]	Y	Y	Y	N	N	Y	N	N	Y	Y	6	Moderate	B
King et al. (2015) [58]	Y	N	N	N	N	N	Y	N	Y	Y	4	Poor	C
Berardelli et al. (2018) [59]	Y	N	Y	N	N	N	Y	N	Y	Y	5	Moderate	B
Buchwitz et al. (2021) [72]	Y	N	Y	N	N	Y	N	N	Y	Y	5	Moderate	B
Rios Romenets et al. (2015) [73]	Y	N	Y	N	N	N	N	Y	Y	Y	5	Moderate	B
Sajatovic et al. (2017) [74]	Y	N	Y	N	N	Y	N	N	Y	Y	5	Moderate	B
Solla et al. (2019) [75]	Y	N	Y	N	N	N	Y	N	Y	Y	5	Moderate	B
Wei et al. (2021) [76]	Y	N	Y	Y	N	Y	Y	N	Y	Y	7	High	B
Sacheli et al. (2019) [81]	Y	N	Y	N	N	Y	Y	Y	Y	Y	7	High	B
Vitale et al. (2024) [84]	Y	N	Y	N	N	Y	Y	Y	Y	Y	7	High	B
**Huntington’s Disease**													
Trinkler et al. (2019) [65]	Y	N	Y	N	N	Y	Y	Y	N	N	7	High	B

## Data Availability

No new data were created or analyzed in this study.

## Supplementary Materials

The following supporting information can be downloaded at: https://www.mdpi.com/article/10.3390/brainsci16070687/s1, Table S1: Reconstructed database search strategy and search strings; Table S2: Full-text articles excluded, with categorized reasons; Table S3: Classification of apathy outcome measures in the 62 included trials (validated apathy-specific vs. proxy/behavioural); Table S4: OCEBM levels of evidence and grades of recommendation framework.

## Abbreviations

The following abbreviations are used in this manuscript:

**Abbreviation**	**Definition**
AARS	Philadelphia Geriatric Center Apparent Affect Rating Scale
AD	Alzheimer’s Disease
AD-RD Mood Scale	Alzheimer’s Disease and Related Disorders Mood Scale
AES	Apathy Evaluation Scale
AES-C	Apathy Evaluation Scale, Clinician-Administered
AI	Apathy Inventory
AiD	Act in Case of Depression
APADEM-NH	Apathy Scale for Institutionalized Patients with Dementia–Nursing Home Version
ARS (PARS)	Philadelphia Geriatric Center Affect Rating Scale
BBS	Berg Balance Scale
BEHAVE-AD	Behavioral Pathology in Alzheimer’s Disease Scale
BI	Barthel Index
BIP	Behavior Observation Scale for Psychogeriatric Inpatients
CMAI	Cohen-Mansfield Agitation Inventory
CSDD	Cornell Scale for Depression in Dementia
DICE	Describe-Investigate-Create-Evaluate
DMPT	Dementia Mood Picture Test
EEG	Electroencephalogram
ERP	Event-Related Potentials
FACS	Functional Assessment of Communication Skills for Adults
G.D.S.	Global Deterioration Scale
GDS	Geriatric Depression Scale
GRGS	Geriatric Resident Goal Scale
HF-rTMS	High-Frequency Repetitive Transcranial Magnetic Stimulation
ICD-10	International Classification of Diseases, 10th Revision
INTERACT	Interventions to Reduce Acute Care Transfers
ITT	Intention to Treat
LPRS	London Psychogeriatric Rating Scale
MCI	Mild Cognitive Impairment
MoCA	Montreal Cognitive Assessment
MOSES	Multidimensional Observational Scale for Elderly Subjects
MSS	Multi-Sensory Stimulation
NAO	Humanoid Social Robot
NIA-AA	National Institute on Aging and Alzheimer’s Association
NICE	National Institute for Health and Care Excellence
NINCDS-ADRDA	National Institute of Neurological and Communicative Disorders and Stroke and the Alzheimer’s Disease and Related Disorders Association
NM Scale	Nishimura Mental State Scale for the Elderly
NPI	Neuropsychiatric Inventory
NPI-NH	Neuropsychiatric Inventory–Nursing Home
NPI-Q	Neuropsychiatric Inventory, Brief Version
OERS	Observed Emotion Rating Scale
PANDA	Parkinson Neuropsychometric Dementia Assessment
PARO	Animal-Shaped Social Robot
PD	Parkinson’s Disease
PDS	Passivity in Dementia Scale
PGCMS	Philadelphia Geriatric Center Morale Scale
QOL-AD	Quality of Life in Alzheimer’s Disease Scale
QUALID	Quality of Life in Late-Stage Dementia
RAID	Rating Anxiety in Dementia Scale
SAS	Starkstein Apathy Scale
SD	Standard Deviation
SES	Social Engagement Scale
SOAPD	Scale for the Observation of Agitation in Persons with Dementia
SPROUT	AI-Assisted Social Prescription Decision System
tDCS	Transcranial Direct Current Stimulation
VAS	Visual Analog Scale
VD	Vascular Dementia
WIB	Well-Being/Ill-Being Scale
