# Supplementary material for: Non-Pharmacological Interventions for Managing Apathy in Older Adults with Neurocognitive Disorders: A Systematic Review of Randomized Controlled Trials
Source: Brain Sci. 2026 Jun 29;16(7):687. doi: 10.3390/brainsci16070687 (PMC13406291; doi:10.3390/brainsci16070687)
Supplement: Supplementary file 1 [file brainsci-16-00687-s001.zip › brainsci-4310037-supplementary.pdf]

Supplementary Material

Non-Pharmacological Interventions for Managing Apathy in Older Adults with Neurocognitive Disorders: A Systematic Review of Randomized Controlled Trials

Table S1. Reconstructed database search strategy and search strings

Search terms were combined with the Boolean operator AND; an English-language limit was applied. Records retrieved are those documented for the primary database search (PubMed/MEDLINE) on 23 March 2026. The same term combinations were applied, with database-appropriate syntax, in PsycInfo, the Cochrane Library, and Google Scholar; reference lists of retrieved articles were hand-searched.

#	Search string (terms combined with Boolean AND)	Records
1	"apathy" AND "neurocognitive disorders"	2,787
2	"apathy" AND "dementia"	2,797
3	"apathy" AND "neurocognitive disorders" AND "treatment"	1,318
4	"apathy" AND "neurocognitive disorders" AND "non-pharmacological treatment"	69
5	"apathy" AND "dementia" AND "non-pharmacological treatment"	87
6	"apathy" AND "Alzheimer's disease" AND "treatment"	696
7	"apathy" AND "Lewy bodies dementia" AND "treatment"	76
8	"apathy" AND "Parkinson's disease" AND "treatment"	711
9	"apathy" AND "Huntington's disease" AND "treatment"	76

Note: Embase, Scopus, and trial registries (e.g., ClinicalTrials.gov) were not searched; this is acknowledged as a limitation in the main text. No automation tools were used in the screening process.

Table S2. Full-text articles excluded, with categorized reasons

Of 256 full-text articles assessed for eligibility, 194 were excluded. Because study-level screening logs were not retained, exclusions are summarized below by primary reason rather than as an itemized study list; the counts correspond to those reported in the PRISMA 2020 flow diagram (Figure 1). Where an article met more than one exclusion criterion, it was assigned to the single most salient reason.

Reason for exclusion	N
Depression not ruled out / not distinguished from apathy	61
Apathy not clearly identified as a target or outcome	26
Insufficient or unextractable data on apathy	34
Pharmacological co-intervention confounding the effect	55
Non-randomized design (quasi-experimental or stepped wedge)	8
Planned study / protocol only (no results)	1
No control group or no randomization	4
Not a systematic review (where a review was sought)	4
Apathy not measured (outcome was another construct, e.g., global cognition)	1
**Total excluded**	**194**

Note: The studies excluded under the design criterion used non-randomized designs (quasi-experimental or stepped-wedge); several reports excluded for other reasons (e.g., Camberg 1999, van Weert 2005, Leontjevas 2013, Oguro 2014, Pereira 2025, Sampaio 2021) are cited in the main text for context, and Huang 2025 is counted under "apathy not measured." Randomized crossover trials, in contrast, were eligible and were retained in the narrative synthesis: two such trials (Kolanowski et al. 2005 and Moyle et al. 2013) appear in Table 1, flagged as crossover and interpreted as within-subject (between-condition) comparisons with carry-over considered (see Section 2.2).

Table S3. Classification of apathy outcome measures in the 62 included trials (validated apathy-specific vs. proxy/behavioural).

Study	Apathy outcome measure	Measure type
Baker et al. (2001) [24]	INTERACT / BRS / BMD (behaviour observation)	Proxy / behavioural
Cott et al. (2002) [25]	Engagement & communication (FACS, LPRS)	Proxy / behavioural
Schrijnemaekers et al. (2002) [26]	Dutch Behavior Observation Scale	Proxy / behavioural
Baker et al. (2003) [27]	Behaviour-observation scales (BRS/BMD)	Proxy / behavioural
Politis et al. (2004) [28]	NPI – apathy domain	Validated apathy-specific
Chapman et al. (2004) [29]	NPI – apathy domain	Validated apathy-specific
Lai et al. (2004) [30]	Social engagement & well-being (SES, WIB)	Proxy / behavioural
Finnema et al. (2005) [32]	BIP (behaviour observation)	Proxy / behavioural
Kolanowski et al. (2005) [22]	Passivity in Dementia Scale (PDS)	Proxy / behavioural
Holmes et al. (2006) [33]	Dementia Care Mapping (DCM)	Proxy / behavioural
Staal et al. (2007) [34]	Assessment of Negative Symptoms in AD	Proxy / behavioural
Tadaka and Kanagawa (2007) [35]	MOSES (observation scale)	Proxy / behavioural
Gitlin et al. (2008) [36]	Activity-engagement (5-item, investigator)	Proxy / behavioural
Raglio et al. (2008) [37]	NPI – apathy domain	Validated apathy-specific
Tappen and Williams (2009) [38]	AD-RD Mood Scale	Proxy / behavioural
Hsieh et al. (2010) [39]	Apathy Evaluation Scale – Clinician (AES-C)	Validated apathy-specific
Lam et al. (2010) [40]	NPI – apathy domain	Validated apathy-specific
Niu et al. (2010) [41]	NPI – apathy domain	Validated apathy-specific
Raglio et al. (2010) [42]	NPI – apathy domain	Validated apathy-specific
Ferrero-Arias et al. (2011) [43]	NPI / DAIR	Validated apathy-specific
Hattori et al. (2011) [44]	Apathy Scale	Validated apathy-specific
Kolanowski et al. (2011) [45]	Passivity & engagement (PDS)	Proxy / behavioural
Maci et al. (2012) [46]	Apathy Evaluation Scale (AES)	Validated apathy-specific

Moyle et al. (2013) [23]	Apathy Evaluation Scale (AES) [crossover]	Validated apathy-specific
Suemoto et al. (2014) [82]	Apathy Evaluation Scale (AES)	Validated apathy-specific
Cugusi et al. (2015) [56]	Starkstein Apathy Scale (short)	Validated apathy-specific
Friedmann et al. (2015) [60]	Apathy Evaluation Scale (AES, Zimmerman short)	Validated apathy-specific
Hashimoto et al. (2015) [57]	Apathy Evaluation Scale (AES)	Validated apathy-specific
King et al. (2015) [58]	Lille Apathy Rating Scale (LARS)	Validated apathy-specific
Rios Romenets et al. (2015) [73]	Apathy Evaluation Scale (AES)	Validated apathy-specific
Telenius et al. (2015) [47]	NPI-Q – apathy	Validated apathy-specific
Treusch et al. (2015) [48]	AES / NPI	Validated apathy-specific
Valenti Soler et al. (2015) [49]	APADEM-NH / Apathy Inventory (AI)	Validated apathy-specific
Amieva et al. (2016) [50]	NPI / Apathy Inventory	Validated apathy-specific
Di Domenico et al. (2016) [51]	Apathy Evaluation Scale (AES)	Validated apathy-specific
Manera et al. (2016) [53]	Apathy Inventory (AI)	Validated apathy-specific
Rajkumar et al. (2016) [54]	NPI-NH – apathy	Validated apathy-specific
Sánchez et al. (2016) [55]	NPI – apathy domain	Validated apathy-specific
Ikemata and Momose (2017) [52]	NPI-NH – apathy	Validated apathy-specific
Sajatovic et al. (2017) [74]	Apathy Evaluation Scale (AES)	Validated apathy-specific
Berardelli et al. (2018) [59]	Apathy Evaluation Scale (AES)	Validated apathy-specific
Schall et al. (2018) [63]	NPI – apathy domain	Validated apathy-specific
Tang et al. (2018) [62]	Apathy Evaluation Scale (AES)	Validated apathy-specific
Balzotti et al. (2019) [61]	NPI – apathy domain	Validated apathy-specific
İnel Manav and Simsek (2019) [64]	Apathy Rating Scale (ARS)	Validated apathy-specific
Sacheli et al. (2019) [81]	Apathy Evaluation Scale (AES)	Validated apathy-specific
Solla et al. (2019) [75]	Starkstein Apathy Scale (SAS)	Validated apathy-specific
Trinkler et al. (2019) [65]	Lille Apathy Rating Scale (LARS)	Validated apathy-specific

Verkaik et al. (2019) [83]	Apathy Evaluation Scale (AES)	Validated apathy-specific
Padala et al. (2020) [68]	Apathy Evaluation Scale – Clinician (AES-C)	Validated apathy-specific
Buchwitz et al. (2021) [72]	Apathy Evaluation Scale (AES)	Validated apathy-specific
Oliveira et al. (2021) [79]	NPI-C – apathy	Validated apathy-specific
Robert et al. (2021) [66]	NPI / Apathy Inventory	Validated apathy-specific
Santagata et al. (2021) [80]	NPI – apathy domain	Validated apathy-specific
Wei et al. (2021) [76]	Starkstein Apathy Scale (SAS)	Validated apathy-specific
Yang et al. (2021) [67]	Apathy Evaluation Scale – Informant (AES-I)	Validated apathy-specific
O'Sullivan et al. (2022) [78]	Apathy Evaluation Scale – Informant (AES-I)	Validated apathy-specific
Vitale et al. (2024) [84]	Apathy Evaluation Scale (AES)	Validated apathy-specific
Yang et al. (2024) [77]	Apathy Evaluation Scale (AES)	Validated apathy-specific
Li et al. (2025) [70]	Chinese NPI (CNPI) – apathy domain	Validated apathy-specific
Yang et al. (2025) [71]	NPI-Q – apathy domain	Validated apathy-specific
Zhuo et al. (2025) [69]	Apathy Evaluation Scale – Informant (AES-I)	Validated apathy-specific

Note: 49 of the 62 trials used a validated apathy-specific instrument; 13 relied on proxy or broader behavioral measures. Classification is based on the primary apathy outcome reported in Table 1.

Table S4. OCEBM levels of evidence and grades of recommendation framework

The Oxford Centre for Evidence-Based Medicine (OCEBM) levels-of-evidence and grades-of-recommendation framework used to appraise the included trials is provided here as a methodological reference. Adapted from the Oxford Centre for Evidence-Based Medicine (OCEBM) Levels of Evidence (https://www.cebm.ox.ac.uk/resources/levels-of-evidence).

Level	Explanation
**Levels of evidence**	
**1**	One or more RCTs (or systematic review of trials) of sufficient size to ensure a low risk of false-positive or false-negative results (narrow confidence interval).
**2**	Good quality cohort studies or low-quality RCT (e.g., too small, <80% follow-up).
**3**	Case–control studies, including systematic reviews of case–control studies.
**4**	Case series and poor quality cohort and case–control studies.
**5**	Expert opinion without explicit critical appraisal or based on physiology, bench research, or “first principles.”
**Grades / strengths of recommendation**	
**A**	Consistent level 1 studies.
**B**	Consistent level 2 or 3 studies or extrapolations from level 1 studies.
**C**	Level 4 studies or extrapolations from level 2 or 3 studies.
**D**	Level 5 evidence or troublingly inconsistent or inconclusive studies of any level.

Abbreviations: OCEBM, Oxford Centre for Evidence-Based Medicine; RCT, randomized controlled trial. Summarized from OCEBM (2009). Reproduced for methodological reference.
